# Genetic differences according to onset age and lung function in asthma: A cluster analysis

**DOI:** 10.1002/clt2.12282

**Published:** 2023-07-14

**Authors:** Han‐Kyul Kim, Ji‐One Kang, Ji Eun Lim, Tae‐Woong Ha, Hae Un Jung, Won Jun Lee, Dong Jun Kim, Eun Ju Baek, Ian M. Adcock, Kian Fan Chung, Tae‐Bum Kim, Bermseok Oh

**Affiliations:** ^1^ Department of Biochemistry and Molecular Biology School of Medicine Kyung Hee University Seoul Korea; ^2^ Department of Biomedical Science Graduate School Kyung Hee University Seoul Korea; ^3^ Mendel Seoul Korea; ^4^ The National Heart and Lung Institute Imperial College London UK; ^5^ Department of Allergy and Clinical Immunology Asan Medical Center University of Ulsan College of Medicine Seoul Korea

**Keywords:** asthma, cluster analysis, genome‐wide association study

## Abstract

**Background:**

The extent of differences between genetic risks associated with various asthma subtypes is still unknown. To better understand the heterogeneity of asthma, we employed an unsupervised method to identify genetic variants specifically associated with asthma subtypes. Our goal was to gain insight into the genetic basis of asthma.

**Methods:**

In this study, we utilized the UK Biobank dataset to select asthma patients (All asthma, *n* = 50,517) and controls (*n* = 283,410). We excluded 14,431 individuals who had no information on predicted values of forced expiratory volume in one second percent (FEV1%) and onset age, resulting in a final total of 36,086 asthma cases. We conducted k‐means clustering based on asthma onset age and predicted FEV1% using these samples (*n* = 36,086). Cluster‐specific genome‐wide association studies were then performed, and heritability was estimated via linkage disequilibrium score regression. To further investigate the pathophysiology, we conducted eQTL analysis with GTEx and gene‐set enrichment analysis with FUMA.

**Results:**

Clustering resulted in four distinct clusters: early onset asthma^normalLF^ (early onset with normal lung function, *n* = 8172), early onset asthma^reducedLF^ (early onset with reduced lung function, *n* = 8925), late‐onset asthma^normalLF^ (late‐onset with normal lung function, *n* = 12,481), and late‐onset asthma^reducedLF^ (late‐onset with reduced lung function, *n* = 6508). Our GWASs in four clusters and in All asthma sample identified 5 novel loci, 14 novel signals, and 51 cluster‐specific signals. Among clusters, early onset asthma^normalLF^ and late‐onset asthma^reducedLF^ were the least correlated (*r*_*g*_ = 0.37). Early onset asthma^reducedLF^ showed the highest heritability explained by common variants (*h* ^*2*^ = 0.212) and was associated with the largest number of variants (71 single nucleotide polymorphisms). Further, the pathway analysis conducted through eQTL and gene‐set enrichment analysis showed that the worsening of symptoms in early onset asthma correlated with lymphocyte activation, pathogen recognition, cytokine receptor activation, and lymphocyte differentiation.

**Conclusions:**

Our findings suggest that early onset asthma^reducedLF^ was the most genetically predisposed cluster, and that asthma clusters with reduced lung function were genetically distinct from clusters with normal lung function. Our study revealed the genetic variation between clusters that were segmented based on onset age and lung function, providing an important clue for the genetic mechanism of asthma heterogeneity.

## 1 INTRODUCTION

Asthma is a complex respiratory disease characterized by nonspecific bronchial hyperresponsiveness and airway inflammation, and it exhibits a wide range of clinical and pathophysiological features.^1^, ^2^, ^3^, ^4^, ^5^, ^6^ As such, defining asthma subtypes via genetic approaches can help improve our understanding of the disease and lead to better management and treatment strategies.^2^

Severe asthma, in particular, poses a significant economic burden on the healthcare system, and pharmacological management of severe asthma remains a major challenge.^7^ Antibody‐based therapies targeting the T‐helper type 2 (Th2) inflammation pathway have been prescribed for patients with severe asthma^8^; however, the heterogeneous nature of asthma emphasizes the need for genetic studies to identify subtype‐specific targets.^9^

Previous studies have attempted to dissect asthma subtypes using various criteria and have identified several genetic associations.^10^ For instance, Ferreira et al. studied the genetic variation that was influenced by the onset age of asthma and identified a specific association between early onset asthma and the *17q21* locus.^11^ Another study conducted by Shrine et al. specifically studied genetic risk components of moderate‐to‐severe asthma that were defined by the appropriate medication or the diagnosis by a doctor and identified three loci associated with moderate‐to‐severe asthma, including signals in *KIAA1109*, *MUC5AC*, and *GATA3*.^12^ Although these studies have detected a number of genome‐wide significant association signals, they have been limited by insufficient phenotypic dissection in explaining subtype‐specific characteristics of asthma. On the other hand, Siroux et al. dissected asthma subtypes into four clusters in an unsupervised manner using the latent class analysis and performed a genetic association analysis on each cluster.^9^ However, this clustering study identified only two genome‐wide significant associations, indicating limited power due to the small sample size of 3001 asthma cases.^9^ Therefore, with a larger sample size, the dissection of asthmatic phenotypes into various subtypes could better reveal the heterogeneous characteristics of asthma that could be explained by genetic variations.

In this study, we utilized the large sample size of asthma patients available in the UK Biobank to classify asthma subtypes through k‐means clustering based on two important variables: onset age and predicted FEV1% values. The primary objectives of our investigation were to identify (i) the differences in clinical phenotypes among asthma clusters, (ii) the specific genetic association signals in asthma clusters by performing cluster‐specific genome‐wide association studies (GWAS), and (iii) the genetic variations among clusters through heritability, genetic correlation, and gene‐set enrichment analysis.

## 2 MATERIALS AND METHODS

### 2.1 Study population for the discovery set

The UK Biobank recruited a total of 488,266 participants from 2006 to 2010.^13^ Ethical approval for data collection from participants was obtained from the North West Multicentre Research Ethics Committee, the National Information Governance Board for Health & Social Care, and the Community Health Index Advisory Group. The UK Biobank possesses a generic Research Tissue Bank approval from the National Research Ethics Service, which allows researchers to conduct studies using UKB data without obtaining additional ethical approvals. Access to the UK Biobank data was granted under the Application Reference Number 56987.

Out of the 488,266 genotyped samples, 66,982 samples were excluded as they were non‐white British or Irish (ethnic background, data field 21000 coded with 1 or 1001 or 1002) or genetically related (data field 22012 above 0.25), resulting in a final sample size of 421,284 (Supplementary Table S29). Among these, asthma cases (All asthma, *n* = 50,517) were defined as individuals diagnosed with asthma by a doctor (data field 22127 and 6152) or those who had reported their onset age (data field 3786). For the clustering analysis, we utilized information on predicted FEV1% and asthma onset age from the UK Biobank dataset. Predicted FEV1% was calculated based on the FEV1 value (data field 3063), using the NHANES III spirometry Caucasian reference panel.^14^ Individuals with heights outside the range of 147–207 cm in males and 145–189 cm in females were excluded from the reference panel. For clustering, asthma cases with available information on predicted FEV1% and onset age (*n* = 36,086) were selected from the 50,517 samples, as data on predicted FEV1% were missing or had values over 200% or under 10% for the remaining cases. Controls (*n* = 283,410) were individuals who had not been diagnosed with bronchitis, emphysema, asthma, rhinitis, eczema, and allergy by a doctor (data field 22127 and 6152) and had not used any asthmatic medications described in Supplementary Table S1. The study design for population selection is presented in Supplementary Figure S2.

### 2.2 Study population for the replication set

To replicate the test using the unused samples of the UK Biobank, we retained 66,982 multi‐ethnic individuals, after excluding 421,284 samples that were used in the discovery set, among the total 488,266 UK Biobank samples. We further excluded 2398 individuals who were genetically related (data field 22012 above 0.25), leaving 64,584 unrelated participants for the replication analysis.

Given the limited sample size of our replication set, we performed a meta‐analysis of the unused UK Biobank samples and the moderate‐to‐severe asthma sample set from the study by Shrine et al. (2019).^12^ We used the stage 1 asthma samples from the Genetics of Asthma Severity and Phenotypes initiative and the Unbiased BIOmarkers in PREDiction of respiratory disease outcome project, both of which were included in the study by Shrine et al. (2019).^12^ To avoid overlapping of samples, we excluded 30,330 phase 1 UK Biobank samples from the 64,584 remaining samples.^12^ Of the 34,254 remaining samples, 12,849 individuals were white British (data field 21000: 1 or 1001 or 1002 or 1003). We selected 1361 asthma cases (data field 20002: non‐cancer illness code, self‐reported) and 8246 controls who had not been diagnosed with bronchitis, emphysema, asthma, rhinitis, eczema, and allergy by a doctor (data field 6152: blood clot, DVT, bronchitis, emphysema, asthma, rhinitis, eczema, and allergy diagnosed by a doctor and medication use). The meta‐analysis was performed using association results from the unused UK Biobank samples (1361 cases and 8246 controls) and the moderate‐to‐severe sample set from Shrine et al. (2019) (25,675 controls and 5135 cases) (Supplementary Figure S1).

To further test the reproducibility of the identified signals, we selected asthma cases (*n* = 3071) from 64,584 unrelated individuals who self‐reported asthma (data field 20002: non‐cancer illness code, self‐reported) after excluding 44,168 non‐white British individuals (data field 21000: 1 or 1001 or 1002 or 1003). For clustering analysis, we selected asthma cases with information on asthma onset age and predicted FEV1% (*n* = 2592) to replicate the association with clusters identified in the discovery set. Similar to the discovery set, we divided the asthma cases into four groups using k‐means clustering: 467 in early onset asthma^normalLF^, 794 in early onset asthma^reducedLF^, 522 in late‐onset asthma^normalLF^, and 809 in late‐onset asthma^reducedLF^. We selected 13,285 controls as individuals who had not been diagnosed with bronchitis, emphysema, asthma, rhinitis, eczema, and allergy by a doctor (data field 6152). Due to the small size of the replication set, we performed a meta‐analysis of the association results from both the unused UK Biobank sample clusters and the discovery sample clusters. We then applied three criteria to identify reproducible signals; *P* < 5E‐8 in discovery clusters, *p* < 0.05 in replication clusters, and *p* values from the meta‐analysis less than P from the discovery or replication clusters.

### 2.3 Genotype data

In phase 1 of the UK Biobank (year 2015), the blood samples of 152,611 participants were genotyped using the Affymetrix Axiom (Affymetrx, Santa Clara, CA, USA) UK BiLEVE array.^15^ In phase 2 (year 2017), the later‐generation Affymetrix Axiom UK Biobank array was used to genotype 335,655 samples. Genotyping imputation was performed using the United Kingdom10K Project and 1000 Genome Project Phase 3 reference panels and single nucleotide polymorphisms (SNPs) with an imputation quality score of ≥0.3 were retained.^15^, ^16^ Quality control of SNPs was carried out using PLINK v.1.90 based on the following exclusion criteria: SNPs with missing genotype call rates >0.05, missing sample call rates >0.05, minor allele frequency < 0.01, and *P* for Hardy‐Weinberg equilibrium test <1.00 × 10^−6^. After quality control, a total of 6,209,219 SNPs were retained for further analysis.

### 2.4 Statistical analysis

We utilized bi‐variate data on asthma onset age and predicted FEV1% to perform *k*‐means cluster analysis. Clustering and comparison of clusters were carried out using the SPSS 25 statistical package (SPSS Inc., Chicago, IL, USA). To analyse the differences in baseline characteristics such as lung function, smoking, comorbidity, medication use, and respiratory symptoms among clusters, we employed Kruskal‐Wallis (for continuous variables) and Chi‐square test (for categorical variables).

We conducted logistic regression analysis for GWAS using PLINK v.1.90 to identify cluster‐specific genetic variants while assuming an additive genetic model. We adjusted for age, sex, body mass index (BMI), smoking status, and PC1‐10.^17^ We performed five separate GWASs: one using All asthma cases (*n* = 50,517) and four others for each cluster (early onset asthma^normalLF^, *n* = 8172; early onset asthma^reducedLF^, *n* = 8925; late‐onset asthma^normalLF^, *n* = 12,481; and late‐onset asthma^reducedLF^, *n* = 6508). We used 283,410 control samples for all GWASs. We set the genome‐wide significance threshold to a *p*‐value of 5.00 × 10^−8^. We compared differences in genetic variants between the clusters to identify cluster‐specific genetic associations.

Additionally, to address potential issues of hidden ancestry and unbalanced case‐control, we conducted the SAIGE analysis using our discovery study set as a post‐sensitivity analysis (https://pan.ukbb.broadinstitute.org/).^18^ Since the SAIGE analysis was a post‐sensitivity analysis, we only tested the 163 lead SNPs that were significant in our discovery data set. We used a Bonferroni‐corrected threshold for significance, with a *p*‐value less than 6.13E‐05 (*p* < 6.13E‐05 = 5.00E‐02/163/5).

We used FUMA, a web‐based platform for comprehensive functional mapping of genetic variants, to identify independent SNPs through linkage disequilibrium (LD, *r* ^2^) clumping for a genomic region.^19^ We selected independent and lead SNPs based on the following criteria; *p* < 5.00 × 10^−8^ for genome‐wide significant SNPs, *r* ^2^ ≤ 0.6 (for independent SNPs) and *r* ^2^ ≤ 0.1 (for lead SNPs) within the 250‐Kb flanking region. We performed conditional analysis using GCTA software and examined all SNPs within the 1‐Mb flanking region.

We used the LDSC regression software (LDSC v1.0.1) to calculate genetic correlation and heritability.^20^ We used GWAS summary statistics for each cluster and for other traits from GWASATLAS^21^ (Supplementary Table S2). To account for multiple testing, we used a Bonferroni‐corrected *p*‐value threshold of *p* < 9.09 × 10^−4^ = 0.05/55 to determine the significance of genetic correlations.

### 2.5 Functional annotation

To evaluate the correlation of the identified SNPs with gene expression, we searched for expression quantitative trait loci (eQTL) signals for all lead SNPs among 11 tissues or cells (B cells, whole blood, oesophagus mucosa, lung, lymphocytes, monocytes, natural killer cells, neutrophils, platelets, spleen, and T‐cells) from various eQTL databases, including DICE,^22^ eQTLcatalogue,^23^ eQTLGen,^24^ GTEx/v8,^25^ scRNA eQTLs,^26^ and Westra‐cis‐eQTL.^27^ For sentinel SNPs without information in the eQTL databases, we used proxy SNPs (*r* ^2^ > 0.4) in the 1‐Mb flanking region of lead SNPs.

The gene‐set enrichment analysis was performed using the marker analysis of genomic annotation (MAGMA), integrated in FUMA. We used the *p‐*values of GWAS summary statistics as input for gene‐based analysis via MAGMA,^28^ which tested 19,028 protein‐coding genes with a 20 kb window for each gene. A stringent Bonferroni correction was applied for multiple testing (*p* < 2.63 × 10^−6^ = 0.05/19028). We obtained 15,481 gene sets from various resources, including KEGG, Reactome,^29^ BioCarta, and Gene Ontology (GO) terms, which were integrated into MSigDB version 5.2^30^ and MAGMA for gene‐set enrichment analysis. Tissue specificity analysis was performed on 54 cell‐type‐specific expression profiles obtained from the GTEx portal. Associations of gene sets with asthma clusters were analysed using MAGMA.^28^ For all gene sets, we calculated competitive *p*‐values to test whether the combined effect of genes in a gene set was significantly greater than that of the same number of randomly selected genes. Gene sets that survived the Bonferroni‐correction (*p* < 3.23 × 10^−6^ = 0.05/15481) were reported.

## 3 RESULTS

### 3.1 Clustering analysis with asthma onset age and predicted FEV1%

We conducted *k*‐means clustering on 36,086 asthma patients based on their onset age and predicted FEV1% values. We identified four clusters, and the scatter plot in Supplementary Figure S3 shows the differences in the two variables used for clustering in each cluster. The early onset asthma^normalLF^ (*n* = 8172) and early onset asthma^reducedLF^ (*n* = 8925) had average onset ages of 15.9 and 12.2 years, respectively, while those of late‐onset asthma^normalLF^ (*n* = 12,481) and late‐onset asthma^reducedLF^ (*n* = 6508) were 46.2 and 46.3 years, respectively (Table 1). The average of predicted FEV1% and FEV1/FVC ratio values of early onset asthma^reducedLF^ (73.9% and 68.7%, respectively) and late‐onset asthma^reducedLF^ (66.5% and 67.3%, respectively) were below the normal range, while those of early onset asthma^normalLF^ (100.6% and 75.95%, respectively) and late‐onset asthma^normalLF^ (94.66% and 75.46%, respectively) were within the normal range (Table 1). As expected, early onset asthma clusters had a higher comorbidity rate with allergic diseases such as hay fever or eczema compared to late‐onset clusters (56.5% in early onset asthma^normalLF^ and 53.3% in early onset asthma^reducedLF^ vs. 39.4% in late‐onset asthma^normalLF^ and 33.3% in late‐onset asthma^reducedLF^) (Table 1 and Supplementary Table S27). Eosinophil counts, current smoker ratios, and medication user ratios were higher in the reduced lung function clusters (early onset asthma^reducedLF^ and late‐onset asthma^reducedLF^) than in the normal lung function clusters (early onset asthma^normalLF^ and late‐onset asthma^normalLF^) (Table 1 and Supplementary Table S27). Furthermore, general asthma symptoms such as wheezing or whistling, cough and sputum on most days, and shortness of breath while walking on level ground were more frequent in the reduced lung function clusters than in the normal lung function clusters for the same onset age clusters, and these symptoms were overall more common in late‐onset clusters than in early onset clusters (Table 1 and Supplementary Table S27).

**TABLE 1:** Baseline characteristics of patients with asthma and controls from the UK Biobank.

	Control	All asthma	EON	EOR	LON	LOR	Significance
*N*	283410	50517	8172	8925	12481	6508	
SEX (male, %)	134191 (47.35)	21367 (42.30)	3870 (47.36)	4623 (51.51)	4380 (35.09)	2577 (39.60)	b – g
AGE^b^	57.17 ± 7.93	55.98 ± 8.20	51.86 ± 8.02	54.26 ± 8.37	57.43 ± 7.52	59.19 ± 7.10	b – g
FEV1/FVC ratio (%)^b^	76.26 ± 6.85	73.16 ± 8.35	75.95 ± 16.38	68.70 ± 20.12	75.46 ± 18.23	67.34 ± 22.73	b – g
Predicted FEV1%^b^	93.22 ± 16.44	86.09 ± 18.25	100.6 ± 10.15	73.86 ± 11.70	94.66 ± 10.61	66.46 ± 11.73	b – g
BMI^b^	27.30 ± 4.61	28.08 ± 5.28	27.01 ± 4.53	27.89 ± 5.28	28.10 ± 5.06	29.00 ± 5.74	b – g
Asthma diagnosed age^b^	NA	28.03 ± 20.32	15.85 ± 9.46	12.16 ± 8.41	46.15 ± 9.15	46.29 ± 10.05	b – f
Eosinophil count^b^	0.16 ± 0.12	0.22 ± 0.18	0.21 ± 0.15	0.24 ± 0.18	0.21 ± 0.17	0.24 ± 0.19	b – f
Current smoker (%)^c^ ^,^ ^a^	29299 (10.38)	4514 (8.97)	590 (7.23)	910 (10.23)	670 (5.39)	834 (12.87)	b – g
Previous smoker (%)^c^ ^,^ ^a^	100803 (35.70)	18282 (36.35)	2608 (31.98)	2766 (31.09)	4814 (38.73)	2797 (43.17)	c – g
Pack years of smoking^b^	22.74 ± 18.21	23.03 ± 19.48	16.59 ± 14.25	21.18 ± 18.12	19.69 ± 15.97	28.62 ± 22.28	b – d, f, g
Medication use (%)^c^ ^,^ ^a^	NA	22369 (44.28)	2774 (33.95)	4301 (48.19)	5725 (45.87)	3633 (55.82)	b – d, f, g
Hay fever, rhinitis, or eczema (%)^c^ ^,^ ^a^	0	22963 (44.57)	4613 (56.45)	4758 (53.31)	4913 (39.36)	2170 (33.34)	b – f
Cough on most days (%)^c^ ^,^ ^a^	7292 (10.64)	4128 (27.81)	370 (15.05)	515 (21.69)	1002 (30.56)	462 (33.8)	b – f
Bring up sputum on most days (%)^c^ ^,^ ^a^	4526 (6.61)	2663 (17.94)	260 (10.57)	374 (15.75)	571 (17.41)	318 (23.26)	b – d, f, g
Wheeze or whistling (%)^c^ ^,^ ^a^	30721 (10.84)	30904 (61.18)	4034 (49.36)	5693 (63.78)	7570 (60.65)	4740 (72.82)	b – g
Shortness of breath walking on level ground (%)^c^ ^,^ ^a^	5429 (5.88)	3514 (21.17)	244 (9.03)	584 (19.01)	762 (18.85)	725 (34.36)	b – d, f, g

*Note*: The letters (b – g), indicates significant differences between clusters (*p* < 5.21E‐4 = 0.05/96) (Supplementary Table S27). The letter “b” indicates significant differences between EON and EOR; “c”, EON and LON; “d”, EON and LOR; “e”, EOR and LON; “f”, EOR and LOR; and “g”, LON and LOR.

Abbreviations: EON, early‐onset asthma with normal lung function; EOR, early‐onset with reduced lung function; LON, late‐onset with normal lung function; LOR, late‐onset with reduced lung function.

^a^ The number in parentheses is the ratio of individuals with the indicated phenotypes among participants who reported the variables.

^b^ Kruskal‐Wallis tests for continuous variables and.

^c^ Chi‐squared tests for categorical variables.

### 3.2 Genome‐wide associations in all asthma samples and four clusters

**FIGURE 1:**
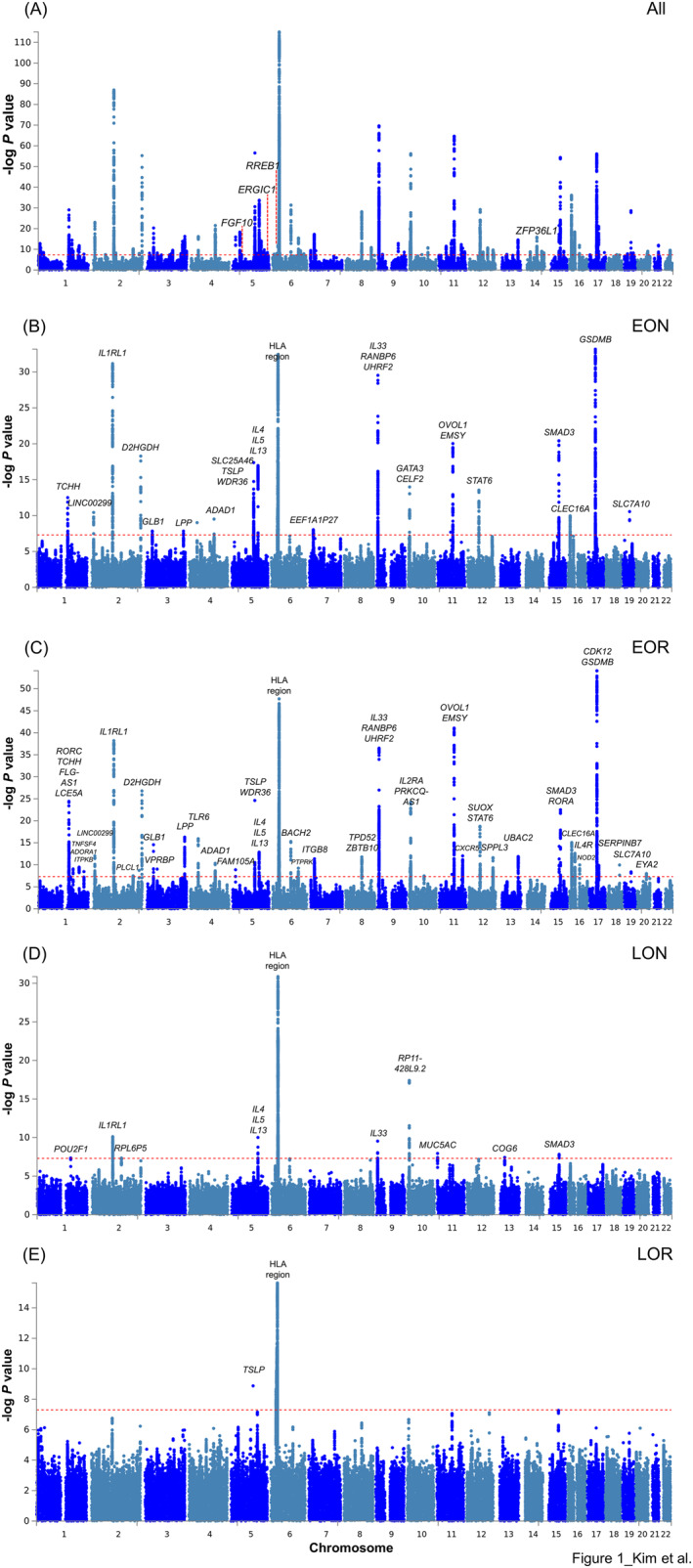
Manhattan plots of GWASs. Manhattan plots were generated via FUMA based on *p*‐values calculated using the logistic regression model using PLINK. (A) All asthma, (B) early onset asthma^normalLF^, (C) early onset asthma^reducedLF^, (D) late‐onset asthma^normalLF^, and (E) late‐onset asthma^reducedLF^. The red dotted line indicates the genome‐wide significance level (*p* < 5.00 × 10^−8^).

All significant genome‐wide association signals from GWASs on All asthma set and four asthma clusters are presented as Manhattan plots (Figure 1) and listed in Supplementary Tables S3–S7. We identified a total of 163 lead SNPs, including 153 SNPs in All asthma, 39 SNPs in early onset asthma^normalLF^, 71 SNPs in early onset asthma^reducedLF^, 16 SNPs in late‐onset asthma^normalLF^, and 5 SNPs in late‐onset asthma^reducedLF^ (Table 2 and Supplementary Table S8).^19^ Among these 163 SNPs, 51 lead SNPs were only significant in one of the four clusters, referred to as cluster‐specific SNPs in our study: 6 SNPs in early onset asthma^normalLF^, 38 SNPs in early onset asthma^reducedLF^, 6 SNPs in late‐onset asthma^normalLF^, and 1 SNP in late‐onset asthma^reducedLF^ (Tables 3 and 4, Supplementary Table S8). Notably, 10 of these 51 cluster‐specific SNPs were not significant in the GWAS on All asthma set: 1 in early onset asthma^normalLF^ (rs2735102, *p* = 1.48 × 10^−10^), 6 in early onset asthma^reducedLF^ (rs4745723, *p* = 3.46 × 10^−8^, rs1811711, *p* = 1.89 × 10^−9^, rs2890664, *p* = 4.92 × 10^−11^, rs539478034, *p* = 2.52 × 10^−12^, rs12964116, *p* = 1.13 × 10^−10^ and rs6094570, *p* = 1.01 × 10^−8^), and 3 in late‐onset asthma^normalLF^ (rs1516145, *p* = 4.59 × 10^−8^, rs28373192, *p* = 1.16 × 10^−8^ and rs9603603, *p* = 3.80 × 10^−8^) (Tables 3 and 4). Additionally, we found that 33 SNPs showed multiple associations in more than one cluster (22 SNPs in two early onset clusters, 8 in three clusters, and 3 in all four clusters; Supplementary Table S8).

**TABLE 2:** Summary of our findings and estimation of cluster heritability.

	Summary		Heritability		
	Lead SNPs	Specific lead SNPs	Heritability	SE	*P*
EON	39	6	0.176	0.024	1.41E‐16
EOR	71	38	0.212	0.028	2.70E‐20
LON	16	6	0.079	0.010	1.03E‐17
LOR	5	1	0.130	0.016	1.45E‐16
All asthma	153	79	0.132	0.013	1.90E‐34
Cluster only^a^	10	‐	‐	‐	‐
Total	163	‐	‐	‐	‐

^a^ Did not satisfy the criteria of ‘GWAS significant in All asthma’ but was significant only in specific clusters.

**TABLE 3:** Summary of novel single nucleotide polymorphisms (SNPs).

rsID	CHR	Position	Nearest gene	Specificity^a^	Effect allele	Novelty^b^	Reported SNP	*R* ^2^	MAF	All asthma	EON	EOR	LON	LOR
										*p* value	*p* value	*p* value	*p* value	*p* value
rs116351845	5	44034122	*FGF10*	All asthma	T	Locus	‐	‐	0.013	**3.36E‐08**	2.92E‐01	5.31E‐03	6.85E‐03	1.70E‐02
rs74696793	5	172277815	*ERGIC1*	All asthma	C	Locus	‐	‐	0.024	**3.33E‐09**	2.48E‐04	1.32E‐03	4.63E‐03	1.19E‐01
rs41302867	6	7240876	*RREB1*	All asthma	A	Locus	‐	‐	0.124	**2.26E‐08**	1.77E‐02	5.22E‐02	4.95E‐03	3.65E‐02
6:32895973:AT:A	6	32895973	*HLA‐DMB*	All asthma	A	Signal	rs11964504	0.002	0.044	**4.29E‐09**	6.33E‐01	2.52E‐02	2.74E‐07	1.19E‐02
rs112563428	6	34785079	*UHRF1BP1*	All asthma	T	Signal	rs28522747	0.014	0.117	**2.91E‐08**	2.60E‐01	1.53E‐06	6.30E‐01	1.56E‐02
rs112119265	7	3062629	*CARD11*	All asthma	G	Signal	rs73033536	0.014	0.060	**1.43E‐12**	4.48E‐04	5.41E‐03	4.42E‐03	2.42E‐05
rs17302823	7	22719409	*IL6*	All asthma	G	Signal	rs34880821	0.081	0.220	**2.70E‐08**	9.91E‐05	1.22E‐02	4.83E‐03	1.36E‐01
rs12889006	14	69260563	*ZFP36L1*	All asthma	C	Locus	‐	‐	0.461	**4.33E‐08**	1.45E‐03	3.02E‐06	3.45E‐01	5.44E‐03
rs833914	19	38554364	*SIPA1L3*	All asthma	T	Locus	‐	‐	0.018	**1.33E‐08**	5.80E‐02	7.75E‐02	7.67E‐04	6.86E‐02
rs2735102	6	29912925	*HLA‐A*	EON	G	Signal	rs2517690	0.002	0.128	7.96E‐07	**1.48E‐10**	1.52E‐02	5.88E‐01	3.44E‐01
rs205002	6	32113312	*PRRT1*	EOR	A	Signal	rs204993	0.013	0.331	**7.56E‐18**	6.49E‐08	**3.05E‐12**	7.90E‐05	9.81E‐01
rs4745723	10	75603338	*CAMK2G*	EOR	C	Signal	rs1134777	0.059	0.194	2.20E‐06	7.33E‐01	**3.46E‐08**	4.44E‐01	2.56E‐01
rs3806155	6	32373378	*BTNL2*	LON	T	Signal	rs3117098	0.006	0.037	**3.65E‐21**	4.24E‐04	7.56E‐07	**2.76E‐13**	1.58E‐02
rs113457465	6	32654241	*HLA‐DQB1*	Multiple	C	Signal	rs17843577	0	0.065	**1.10E‐39**	**8.65E‐15**	**1.10E‐13**	**4.94E‐13**	5.29E‐03

*Note*: Genome‐wide significant signals are stated in bold, and the specificity column was determined by whether the genome‐wide significance was satisfied for each study. The SNP reported by the same locus was described on the Reported SNP and the LD value with that SNP was displayed in the *R* ^2^ column. The italic words indicate the name for human genes.

^a^ All asthma, only significant in all asthma; EON‐LOR, Significant for specific clusters only; Multiple, Significant in 3 clusters; Significance of all asthma was not considered when determining cluster specificity.

^b^ Locus, newly discovered locus; Signal, newly discovered signal from reported locus.

**TABLE 4:** Summary of cluster‐specific lead single nucleotide polymorphisms (SNPs).

rsID	CHR	Position	Reported locus	Specificity	Effect allele	Reported SNP	*R* ^2^	MAF	All asthma	EON	EOR	LON	LOR
									*p* value	*p* value	*p* value	*p* value	*p* value
Early onset asthma with reduced lung function specific signals													
rs3828058	1	151786281	*RORC*	EOR	A	rs3828058	1	0.373	3.37E‐11	2.72E‐07	3.66E‐09	9.64E‐02	3.50E‐01
rs11205006	1	152440176	*LCE5A*	EOR	A	rs11205006	1	0.283	**1.70E‐15**	4.27E‐05	**1.70E‐14**	1.65E‐01	3.45E‐01
rs4090390	1	173146921	*TNFSF4*	EOR	A	rs4090390	1	0.240	**1.03E‐09**	6.14E‐05	**8.93E‐10**	7.23E‐01	3.48E‐01
rs12411260	1	203078793	*ADORA1*	EOR	T	rs6683383	0.885	0.321	**1.43E‐11**	1.37E‐03	**4.60E‐10**	5.98E‐02	3.66E‐01
rs3768410	1	226910570	*ITPKB*	EOR	T	rs697852	0.216	0.498	**2.23E‐08**	1.44E‐01	**3.59E‐09**	9.09E‐02	4.42E‐02
rs1318867	2	198880481	*PLCL1*	EOR	A	rs2164068	0.853	0.480	**1.71E‐10**	2.94E‐04	**3.29E‐08**	4.09E‐03	3.34E‐01
rs1811711	2	228670476	*AC073065.3*	EOR	G	rs1811711	1	0.192	6.89E‐06	8.13E‐05	**1.89E‐09**	7.66E‐01	4.11E‐01
rs35570272	3	33047662	*GLB1*	EOR	T	rs35570272	1	0.395	**5.21E‐21**	1.16E‐07	**2.76E‐15**	1.61E‐02	1.29E‐03
rs73078636	3	51441307	*VPRBP*	EOR	A	rs73078636	1	0.127	**6.05E‐09**	7.74E‐05	**9.65E‐10**	1.42E‐01	7.20E‐02
rs11728049	4	38757168	*TLR1*	EOR	G	rs5743618	0.329	0.197	**4.65E‐16**	3.56E‐07	**1.19E‐16**	2.18E‐01	5.04E‐02
rs2890664	4	38826978	*TLR6*	EOR	A	rs5743618	0.131	0.477	7.47E‐08	2.96E‐06	**4.92E‐11**	6.62E‐01	8.82E‐01
rs16903574	5	14610309	*OTULINL*	EOR	G	rs16903574	1	0.068	**2.95E‐15**	5.13E‐07	**1.35E‐09**	1.92E‐01	5.91E‐04
rs115222658	6	32694042	*HLA‐DQB3*	EOR	A	rs9275698	0.115	0.063	**1.03E‐16**	9.33E‐06	**1.57E‐09**	3.69E‐04	3.36E‐02
6:32890908:AAAAC:A	6	32890908	*HLA‐DMB*	EOR	A	rs9275602	0.272	0.290	**9.20E‐16**	1.32E‐06	**6.63E‐13**	1.16E‐01	2.44E‐03
rs13203715	6	32988588	*HLA‐DOA*	EOR	G	rs11964504	0.446	0.113	**3.93E‐15**	3.07E‐07	**4.26E‐08**	3.33E‐02	2.47E‐02
rs9277610	6	33070732	*HLA‐DPB1*	EOR	G	rs3097670	0.96	0.126	**4.81E‐13**	7.93E‐07	**1.29E‐10**	2.22E‐01	4.24E‐02
rs72928038	6	90976768	*BACH2*	EOR	A	rs2325291	0.364	0.178	**1.44E‐24**	2.26E‐03	**5.12E‐16**	1.45E‐06	2.20E‐02
rs41285280	6	128291649	*PTPRK*	EOR	T	rs55743914	0.969	0.238	**4.14E‐16**	6.49E‐06	**6.22E‐09**	9.23E‐04	5.78E‐01
rs34759507	7	20419149	*ITGB8*	EOR	CAA	rs149317277	0.935	0.419	**6.93E‐16**	1.33E‐03	**4.43E‐12**	4.03E‐02	7.88E‐05
rs6473228	8	81299766	*ZBTB10*	EOR	G	rs12543811	0.846	0.365	**1.00E‐28**	1.28E‐05	**4.62E‐12**	4.39E‐05	4.48E‐04
rs12722547	10	6072093	*IL2RA*	EOR	C	rs12722547	1	0.017	**1.24E‐20**	6.34E‐04	**2.68E‐09**	1.34E‐05	6.71E‐04
rs41284471	10	6626214	*PRKCQ‐AS1*	EOR	A	rs41284471	1	0.202	**3.05E‐14**	1.74E‐07	**7.42E‐10**	9.77E‐01	3.21E‐03
rs2025758	10	8841669	*LINC02676*	EOR	C	rs2025758	1	0.457	**1.84E‐19**	1.48E‐05	**1.31E‐08**	2.33E‐05	7.86E‐03
rs77740805	10	9393484	*LINC00709*	EOR	A	rs17406680	0.211	0.040	**3.25E‐11**	2.17E‐02	**2.03E‐10**	3.95E‐03	5.87E‐02
rs12365699	11	118743286	*CXCR5*	EOR	A	rs12365699	1	0.167	**3.32E‐11**	5.26E‐07	**9.38E‐13**	2.58E‐01	9.10E‐01
rs1081975	12	56393337	*SUOX*	EOR	G	rs705700	1	0.178	**5.82E‐21**	2.57E‐03	**1.89E‐19**	1.24E‐05	3.22E‐05
rs539478034	12	121265596	*SPPL3*	EOR	AT	rs625228	0.546	0.414	1.59E‐07	3.49E‐04	**2.52E‐12**	4.93E‐01	5.31E‐01
rs9517640	13	99844712	*UBAC2*	EOR	A	rs59186511	0.955	0.118	**1.09E‐14**	8.83E‐05	**1.45E‐12**	1.58E‐03	2.53E‐01
rs34986765	15	61069201	*RORA*	EOR	C	rs10519068	0.844	0.128	**3.33E‐22**	9.19E‐08	**6.52E‐15**	1.14E‐02	1.52E‐02
rs3785356	16	27349168	*IL4R*	EOR	T	rs3785356	1	0.292	**4.37E‐19**	5.66E‐07	**2.60E‐13**	1.85E‐03	6.24E‐01
rs12324931	16	50790158	*CYLD*	EOR	C	rs2066844	0.895	0.044	**1.28E‐12**	5.11E‐03	**1.09E‐10**	1.55E‐03	1.16E‐02
rs72827105	17	37643376	*CDK12*	EOR	G	rs145835664	0.719	0.172	**3.96E‐15**	1.05E‐06	**6.49E‐13**	1.90E‐01	1.50E‐01
rs117097909	17	38064971	*GSDMB*	EOR	A	rs117097909	1	0.052	**9.52E‐17**	1.77E‐07	**3.96E‐15**	5.65E‐01	2.47E‐01
rs16948048	17	47440466	*AC091180.3*	EOR	G	rs16948048	1	0.369	**5.82E‐21**	4.05E‐06	**1.36E‐10**	2.86E‐04	7.81E‐01
rs12964116	18	61442619	*SERPINB7*	EOR	G	rs12964116	1	0.037	5.96E‐08	2.77E‐05	**1.13E‐10**	9.24E‐01	3.39E‐01
rs6094570	20	45682341	*EYA2*	EOR	G	rs8125525	1	0.265	1.02E‐06	5.90E‐01	**1.01E‐08**	2.14E‐02	6.61E‐01
Other clusters specific signals													
rs79337446	3	33088785	*RAF1*	EON	T	rs79337446	1	0.067	**1.85E‐11**	**1.44E‐08**	8.25E‐07	7.15E‐02	4.30E‐01
rs139671781	5	110426482	*WDR36*	EON	A	rs149205188	1	0.036	**8.43E‐11**	**6.09E‐10**	3.33E‐05	2.73E‐01	6.40E‐03
rs28712631	7	20543689	*ITGB8*	EON	G	rs12531500	1	0.419	**6.60E‐17**	**9.25E‐09**	5.99E‐08	2.66E‐03	1.65E‐02
rs7894791	10	8591369	*GATA3*	EON	A	rs7894791	1	0.412	**3.79E‐15**	**2.12E‐08**	7.80E‐06	3.71E‐03	1.92E‐03
rs10152595	15	67475488	*SMAD3*	EON	G	rs10152595	1	0.246	**7.44E‐25**	**1.61E‐09**	7.81E‐07	5.73E‐07	2.41E‐02
rs1021621	1	167198536	*POU2F1*	LON	G	rs6427082	0.914	0.473	**2.26E‐09**	6.90E‐03	2.99E‐02	**4.55E‐08**	3.16E‐01
rs1516145	2	146144841	*RPL6P5*	LON	A	rs2381712	1	0.482	4.22E‐06	9.75E‐01	6.26E‐02	**4.59E‐08**	2.36E‐02
rs6894249	5	131797547	*IRF1*	LON	G	rs3749833	0.544	0.389	**5.94E‐29**	8.18E‐08	4.08E‐07	**9.81E‐11**	1.25E‐05
rs28373192	11	1157035	*MUC5AC*	LON	C	rs12788104	0.275	0.414	1.78E‐06	1.30E‐02	8.08E‐02	**1.16E‐08**	7.13E‐01
rs9603603	13	40300328	*COG6*	LON	G	rs28635831	0.848	0.365	1.28E‐06	5.73E‐01	3.00E‐02	**3.80E‐08**	8.47E‐01
rs1034326	6	30084446	*TRIM26*	LOR	G	rs1117490	0.251	0.163	**3.46E‐10**	5.32E‐02	8.57E‐05	7.62E‐02	**8.94E‐12**

*Note*: The italic words indicate the name for human genes. The bold values indicate the *p* values that satisfy genome‐wide significance.

The plotted *p*‐values of associated signals showed that early onset asthma^reducedLF^ had more significant association signals with smaller *p*‐values than the other clusters (Supplementary Figure S4). Pairwise plotting of the association *p*‐values within the early onset or late‐onset clusters revealed that early onset asthma^reducedLF^ had more significant *p*‐values than early onset asthma^normalLF^, and late‐onset asthma^normalLF^ had more significant *p*‐values than late‐onset asthma^reducedLF^ (Supplementary Figure S5).

### 3.3 Novel association signals

To identify novel signals among the 163 lead SNPs, we examined the association studies on asthma in the GWAS Catalogue and found 231 SNPs that were previously reported to be associated with asthma (Supplementary Table S10). Out of 163 lead SNPs, 149 SNPs, including 62 sentinel and 87 proxies, have been previously reported.

Of the total 163 lead SNPs, 14 were identified as novel signals that had not been previously reported (Table 3). To confirm the novelty of these SNPs, conditional analysis with GCTA was performed, validating them as previously unidentified (Supplementary Table S9).^31^ Of these 14 SNPs, 5 were located at novel loci for asthma that had not been reported yet (Table 3): rs116351845 near *FGF10* (*p* = 3.36 × 10^−8^ in All asthma), rs74696793 in *ERGIC1* (*p* = 3.33 × 10^−8^ in All asthma), rs41302867 in *RREB1* (*p* = 2.26 × 10^−8^ in All asthma), rs12889006 in *ZFP36L1* (*p* = 4.33 × 10^−8^ in All asthma), and rs833914 in *SIPA1L3* (*p* = 4.33 × 10^−8^ in All asthma). These 5 novel loci SNPs were significant only in All asthma GWAS. Among the remaining 9 SNPs, 4 were cluster‐specific novel SNPs: rs2735102 in *HLA‐H* (*p* = 1.48 × 10^−10^ in early onset asthma^normalLF^), rs205002 in *PRRT1* (*p* = 3.05 × 10^−12^ in early onset asthma^reducedLF^), rs4745723 in *CAMK2G* (*p* = 3.46 × 10^−8^ in early onset asthma^reducedLF^), and rs3806155 in *BTNL2* (*p* = 2.76 × 10^−13^ in late‐onset asthma^normalLF^). A detailed description of functional annotations for these lead SNPs is provided in the Supplementary results.

### 3.4 Validation of association signals and cluster‐specific signals

To replicate the results of all asthma patients, we applied Bonferroni correction based on the number of available signals (114 SNPs) in the replication set, with a significance threshold of *p* < 4.39E‐04 (0.05/114) in the meta‐analysis with the moderate‐to‐severe sample set in Shrine et al.^12^ We checked whether the direction of effect was consistent with our discovery results. Of the 153 SNPs significant in All asthma, 4 SNPs were replicated, and of the 114 available SNPs, 35 SNPs were replicated in the moderate‐to‐severe sample set. In the end, a total of 41 SNPs were replicated in the meta‐analysis of the unused phase 2 UK Biobank set and the moderate‐to‐severe sample set (Supplementary Tables S11 and S12). However, among 14 novel signals, 6 available SNPs were not replicated in this meta‐analysis (Table 3 and Supplementary Tables S11 and S12).

Reproducing GWASs with clusters using summary statistics from previous studies is not feasible. To reproduce GWAS with clusters, we performed k‐means clustering on the unused British and Irish replication set of the UK Biobank (2592 asthma patients) and conducted association analysis in four clusters and controls (*n* = 13,285): 467 early onset asthma^normalLF^, 794 early onset asthma^reducedLF^, 522 late‐onset asthma^normalLF^, and 809 late‐onset asthma^reducedLF^. We conducted a meta‐analysis of the association results from both the unused UK Biobank sample clusters and the discovery sample clusters. A total of 51 reproducible signals were identified after the application of three criteria: *P* < 5E‐8 in discovery clusters, *p* < 0.05 in replication clusters, and *p* values from the meta‐analysis less than P from the discovery or replication clusters. We identified reproducible associations of 9 out of 39 in early onset asthma^normalLF^, 38 out of 72 in early onset asthma^reducedLF^, 2 out of 16 in late‐onset asthma^normalLF^, and 2 out of 5 in late‐onset asthma^reducedLF^ (Supplementary Tables S13 and 14). Among the cluster‐specific novel signals, rs205002 in *PPRT1* was consistently reproducible in early onset asthma^reducedLF^ of the replication set and rs113457465 in *HLA‐DQB1* was reproducible in early onset asthma^normalLF^ of the replication set (Supplementary Table S14).

Despite the relatively small size of the replication set, we observed that more than half of the cluster‐specific SNPs were reproduced in early onset asthma^reducedLF^. Among the lead SNPs specific to early onset asthma^reducedLF^, several important eQTLs were identified. Those eQTLs included signals that are correlated with cell activation, such as *TNFSF4* (rs4090390), *ITPKB* (rs3768410), and *PRKCQ‐AS1* (rs41284471), signals for genes regulating pathogen recognition, such as *CXCR5* (rs12365699), and signals for T cell activation, such as *RORA* (rs34986765) and *RORC* (rs3828058).

We assessed the potential impact of population stratification on our results by calculating the genomic inflation factors (λ_GC_) and LD score regression intercepts as measures of confounding bias.^20^ In our study, genomic inflation factors (λ_GC_) and LD score regression intercepts were 1.33 and 1.070 for all samples, 1.12 and 1.012 for EON, 1.17 and 1.039 for EOR, 1.10 and 1.018 for LON, and 1.09 and 0.998 for LOR cluster, respectively. These results suggest that our study is unlikely to suffer from false‐positive errors due to population stratification.

Additionally, to address potential issues of hidden ancestry and unbalanced case‐control, we conducted the SAIGE analysis using the 163 lead SNPs that were significant in our discovery data set as a post‐sensitivity analysis.^18^ Our results indicated that, out of 163 lead SNPs, a total of 143 lead SNPs satisfied the Bonferroni‐corrected threshold for significance in the SAIGE sensitivity analysis (35 of 39 in EON, 60 of 71 in EOR, 14 of 16 in LON, 3 of 5 in LOR, and 130 of 153 in all asthma data set; see Supplementary Table S28). Therefore, our findings suggest that at least 93.5% of our lead SNPs are unlikely to be affected by false positive errors due to the unbalanced case‐control issue and hidden ancestry.

### 3.5 Heritability and genetic correlation between clusters and other traits

We used LDSC to estimate the heritability of All asthma and four clusters using the summary statistics obtained in this study. The heritability of clusters ranged from 0.079 to 0.212, while that of All asthma set was estimated to be 0.132 (Table 2). Consistent with previous studies, we observed higher heritability in early onset clusters (*h* ^2^ = 0.176 in early onset asthma^normalLF^ and *h* ^2^ = 0.212 in early onset asthma^reducedLF^) compared to late‐onset clusters (*h* ^2^ = 0.079 in late‐onset asthma^normalLF^ and *h* ^2^ = 0.130 in late‐onset asthma^reducedLF^).^11^

**FIGURE 2:**
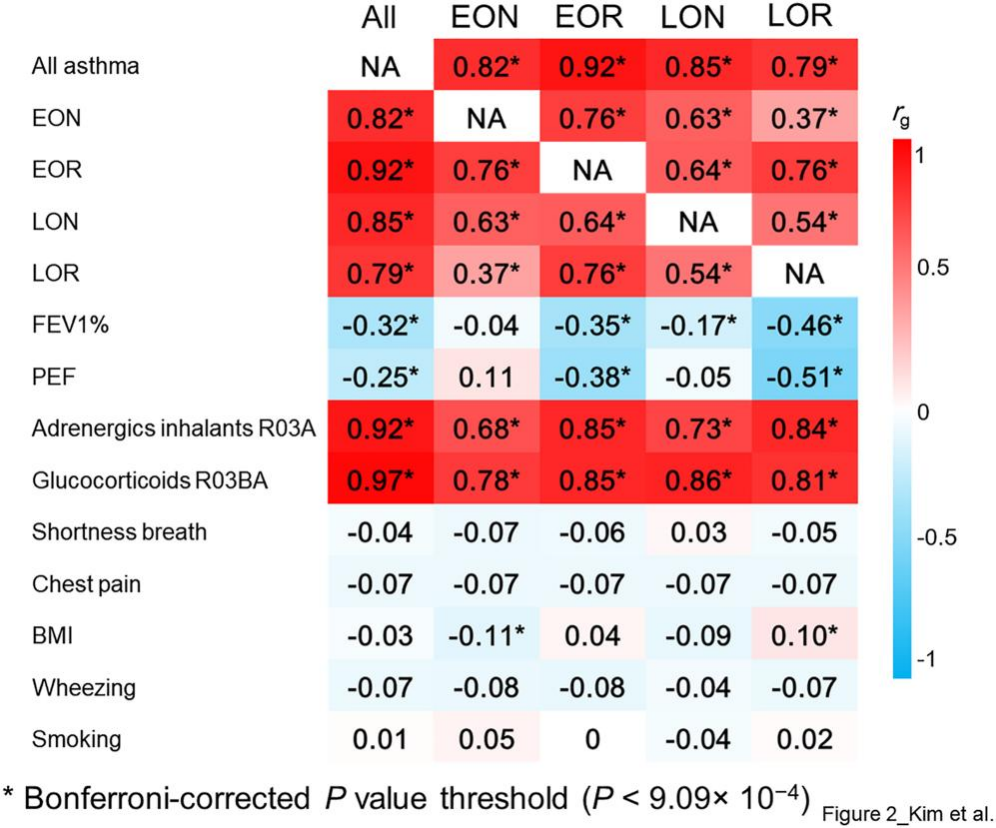
Genetic correlations of asthma clusters within clusters and with other traits. Genetic correlations of clusters within clusters and with nine asthma‐related traits were computed using cross‐trait linkage disequilibrium (LD) score regression. Red and blue indicate positive and negative genetic correlations, respectively, whereas the gradient indicates the strength of the genetic correlations. Sample sizes for the analysis of each trait are provided in Supplementary Table S26.

We utilized LDSC to investigate the degree of genetic risk factor sharing among clusters (Figure 2 and Supplementary Table S15). The clusters exhibited somewhat similar genetic correlations with All asthma: early onset asthma^normalLF^ = 0.82, early onset asthma^reducedLF^ = 0.92, late‐onset asthma^normalLF^ = 0.85, and late‐onset asthma^reducedLF^ = 0.79. We also examined the extent of genetic risk factor sharing between pairwise clusters. The highest genetic correlations were observed between late‐onset asthma^normalLF^ and early onset asthma^reducedLF^ (*r*_*g*_ = 0.76), while the lowest was between early onset asthma^normalLF^ and late‐onset asthma^reducedLF^ (*r*_*g*_ = 0.37). The genetic correlation between late‐onset clusters (late‐onset asthma^normalLF^ and late‐onset asthma^reducedLF^, *r*_*g*_ = 0.54) was lower than that between early onset clusters (early onset asthma^normalLF^ and early onset asthma^reducedLF^, *r*_*g*_ = 0.76), and the genetic correlation between reduced lung function clusters was higher (early onset asthma^reducedLF^ and late‐onset asthma^reducedLF^, *r*_*g*_ = 0.76) than that between normal lung function clusters (early onset asthma^normalLF^ and late‐onset asthma^normalLF^, *r*_*g*_ = 0.63).

We also examined the genetic correlations of clusters with nine asthma‐related traits. As expected, we observed a significant negative genetic correlation between lung function traits, such as FEV1/FVC ratio and PEF only in reduced lung function clusters (early onset asthma^reducedLF^ *r*_*g*_ = −0.35 and late‐onset asthma^reducedLF^ *r*_*g*_ = −0.46 with FEV1/FVC ratio; early onset asthma^reducedLF^ *r*_*g*_ = −0.38, and late‐onset asthma^reducedLF^ *r*_*g*_ = −0.51 with PEF). Interestingly, we found a significant genetic correlation with BMI in early onset asthma^normalLF^ and late‐onset asthma^reducedLF^ but in opposite directions (early onset asthma^normalLF^ *r*_*g*_ = −0.11 and late‐onset asthma^reducedLF^ *r*_*g*_ = 0.10). No significant genetic correlations were observed with other asthma‐related traits, such as shortness of breath, chest pain, wheezing, and smoking.

### 3.6 Gene‐set enrichment analysis for asthma clusters

Tissue specificity and gene analysis of cluster GWAS signals were investigated through gene‐based GWAS. A detailed description of these analyses is provided in the ‘Supplementary results’ section. Gene set analyses were conducted using KEGG, Reactome, BioCarta, and GO data from MSigDB, and the results are presented in Tables S16, S17, and S18.^30^ Based on multiple correction (*p* < 3.23 × 10^−6^), 11 gene sets were identified as significant in early onset asthma^normalLF^, 54 in early onset asthma^reducedLF^, and none in either late‐onset asthma^normalLF^ or late‐onset asthma^reducedLF^. The significant gene sets for biological processes were visualised using GOView (Supplementary Figure S6). The gene‐wide significance level was defined as a *p* value of <2.63 × 10‐6 (0.05/19,018 genes), represented as a red line in gene‐based Manhattan plots (Supplementary Figure S7), and the number of significant genes is shown in Venn diagrams (Supplementary Figure S8). We also examined the tissue specificity of cluster‐significant genes using MAGMA (Supplementary Figure S9).

Many gene sets identified in early onset asthma^normalLF^ (6 out of 11 gene sets) were also significant in early onset asthma^reducedLF^, including cytokine‐cytokine receptor interaction, negative regulation of T‐helper 1 type immune response, type 2 immune response, regulation of type 2 immune response, regulation of isotype switching to IgE isotypes, and interleukin 1 receptor activity. Among the early onset asthma^reducedLF^‐specific gene sets, three categories were noteworthy: leucocyte differentiation, lymphocyte activation, and cytokine production. The category of leucocyte differentiation contained gene sets related to T‐cell differentiation, including T‐cell differentiation involved in immune response (*p* = 1.21 × 10^−7^), alpha beta T‐cell differentiation (*p* = 5.76 × 10^−11^), and regulation of T cell differentiation (*p* = 1.82 × 10^−8^). The category of leucocyte activation contained gene sets related to B‐ and T‐cell activation, including the positive regulation of B cell activation (*p* = 7.05 × 10^−7^) and CD4‐positive alpha beta T‐cell activation (*p* = 1.88 × 10^−9^). The category of cytokine production contained the gene set associated with transforming growth factor beta activation (*p* = 1.42 × 10^−7^).

## 4 DISCUSSION

Applying *k*‐means clustering to data at onset age and predicted FEV1% from 36,086 asthma patients in the UK Biobank yielded four asthma clusters. The cluster GWASs identified 163 lead SNPs, of which 14 represent novel association signals, and 51 were specific to the clusters.

We propose that the heterogeneity of asthma has impeded the identification of genetic factors in previous asthma GWASs. Therefore, we first classified patients with asthma into clusters before performing GWAS, which allowed us to identify 10 additional lead SNPs with genome‐wide significance in specific clusters that were not significant in All asthma. Among these SNPs, two (rs2735102 in early onset asthma^normalLF^ and rs4745723 in early onset asthma^reducedLF^) have not been reported before.

Through these GWASs, we identified 14 novel signals, of which 5 (rs116351845, rs74696793, rs41302867, rs12889006, and rs833914) had not been previously reported as asthma loci. Additionally, we discovered 9 novel signals from loci previously associated with asthma. A detailed description of these SNPs is provided in the ‘Supplementary Discussion,’ including their genetic location, the nearest or within gene, gene function, and related disease. For example, *FGF10*, the nearest gene to rs116351545, has been implicated in various lung diseases such as idiopathic pulmonary fibrosis, bronchopulmonary dysplasia, and chronic obstructive pulmonary disease and lung function.^32^, ^33^ Furthermore, we found that the SNP rs116351845 was the eQTL of *NNT* (Nicotinamide Nucleotide Transhydrogenase) that was associated with the response to bronchodilator.^34^ *RREB1*, where the SNP rs41302867 is located, has been associated with the FEV1/FVC ratio^35^ and leukocyte count.^36^ Similarly, *ZPF36L1*, where the SNP rs12889006 is located, reportedly ensures functional immune cell formations.^37^, ^38^ *SIPAIL3*, within which the SNP rs833914 is located, is expressed in monocytes and B cells according to ProteomicsDB and is involved in haematopoietic progenitor cell differentiation.^39^, ^40^

Besides the novel signals, this study identified 51 cluster‐specific signals, most of which belonged to early onset asthma^reducedLF^ (38 lead SNPs) (Table 4). Investigation of eQTL genes for early onset asthma^reducedLF^‐specific lead SNPs revealed genes related to signalling for cell activation, including *TNFSF4* (rs4090390), *ITPKB* (rs3768410), *PLCL1* (rs1318867), *BACH2* (rs72928038), *THEMIS* (rs41285280), *ZBTB10* (rs6473228), *PRKCQ‐AS1* (rs41284471), and *UBAC2* (rs9517640). In addition, genes regulating pathogen recognition, such as *TLR1, TLR6, TLR10* (rs11728049, rs2890664), and *NOD2* (rs12324931), and cytokine receptors, such as *CCR4* (rs35570272), and genes related to T cell differentiation, such as *IL4R* (rs3785356), *RORA* (rs34986765), and *RORC* (rs3828058), were identified. Among these early onset asthma^reducedLF^‐specific signals, *TNFSF4* (rs4090390), *ITPKB* (rs3768410), *CXCR5* (rs12365699), *RORA* (rs34986765), and *RORC* (rs3828058) were also found to be reproducible in a replication test.

In addition to the first two objectives of resolving the heterogeneity of asthma and identifying novel and cluster‐specific genetic signals, this study had a third objective of elucidating genetic differences in asthma clusters. To achieve this, we performed heritability estimation, genetic correlation analysis, and a comparison of association signals among clusters. Late‐onset asthma^reducedLF^ showed the highest negative correlation with lung function traits and had the highest percentage of respiratory symptoms, such as cough, sputum production, wheezing, and shortness of breath during walking at ground level (Table 1). It also showed the highest percentage of smoking status and medication use. Moreover, late‐onset asthma^reducedLF^ had the lowest number of lead SNPs and shared the least number of lead SNPs and genes with other clusters. These findings suggest that the difference in *p*‐values between late‐onset asthma^normalLF^ and late‐onset asthma^reducedLF^ was greater than that observed between early onset asthma^normalLF^ and early onset asthma^reducedLF^. These results may indicate a distinction between late‐onset asthma^reducedLF^ and other clusters, highlighting the heterogeneity between late‐onset asthma^normalLF^ and late‐onset asthma^reducedLF^ and the similarity between early onset asthma^normalLF^ and early onset asthma^reducedLF^. Moreover, the heritability explained by common variants was highest in early onset asthma^reducedLF^ (*h* ^*2*^ = 0.212), as was the number of associated lead SNPs and genes for each cluster (71 SNPs and 167 genes), and the number of significant gene sets for pathways (54 for gene set).

The box plot (Supplementary Figure S4A) and the volcano plots (Supplementary Figure S4B) clearly show that the odds ratio signals and *p*‐values in early onset asthma^normalLF^ and early onset asthma^reducedLF^ were higher than those in late‐onset asthma^normalLF^ and late‐onset asthma^reducedLF^, with the highest values observed in early onset asthma^reducedLF^. These results suggest that asthma patients in the early onset asthma^reducedLF^ were more likely to be genetically predisposed to asthma.

Shared gene sets between early onset asthma^normalLF^ and early onset asthma^reducedLF^ included cytokine‐cytokine receptor interaction, negative regulation of T‐helper 1 type immune response, type 2 immune response, regulation of type 2 immune response, regulation of isotype switching to IgE isotypes, and interleukin 1 receptor activity. These gene sets suggest that they are associated with the incidence of early onset asthma in both early onset asthma^normalLF^ and early onset asthma^reducedLF^. Negative regulation of Th1 following Th2 activation and IgE binding to myeloid cells (eosinophils and mast cells) are well‐known mechanisms of the immune response in asthma.^8^

We have also identified gene sets specific to early onset asthma^reducedLF^, including leukocyte differentiation, lymphocyte activation, and cytokine production (Supplementary Table S19). These results are supported by examining early onset asthma^reducedLF^‐specific lead SNPs corresponding to eQTL genes and functional annotations (Supplementary Tables S20, S21, and S22). Among the early onset asthma^reducedLF^‐specific eQTL genes (Supplementary Tables S23 and S24), several are related to lymphocyte differentiation, activation, and proliferation. For instance, *ITPKB* and *RORC*, eQTL genes carrying early onset asthma^reducedLF^‐specific lead SNPs rs3768410 and rs3828058, respectively, showed early onset asthma^reducedLF^‐specific gene‐based significances in MAGMA analysis (Supplementary Tables S25 and S26) and are involved in alpha beta T‐cell activation and differentiation. Therefore, we speculate that once early onset asthma develops, further the worsening of symptoms (such as reduced lung function as in early onset asthma^reducedLF^) may progress via the activated status of lymphocytes, which may be due to the genetic profile detected in early onset asthma^reducedLF^ patients.

This study had several limitations. Firstly, the lack of replication of the 14 novel association signals was a significant limitation. Secondly, the absence of gene sets for the two late‐onset clusters, due to the inability to identify sufficient genes for gene‐set enrichment analysis, was a limitation. A larger sample size of late‐onset asthma patients with better phenotype characterization may be required to identify gene sets related to late‐onset development. Additionally, other factors such as the exposome may play a more significant role in late‐onset asthma development. Thirdly, there were limited clinical measurements of asthma patients in the UK Biobank due to the use of only two features for clustering analysis. This is due to the nature of the UK Biobank, which is a population‐based cohort that measures general clinical measurements of participants.

In conclusion, we have identified 14 novel lead SNPs, including five new loci from the asthma cluster GWASs. Examination of early onset asthma^reducedLF^‐specific lead SNPs revealed four possible pathways involved in the worsening of symptoms in early onset asthma: lymphocyte activation, pathogen recognition, cytokine receptor, and lymphocyte differentiation, which was further supported by gene‐set enrichment analysis results. Additionally, early onset asthma^reducedLF^ was found to be the most genetically predisposed cluster, whereas late‐onset asthma^reducedLF^ was distinct from the other clusters. Despite the study's limitations, the findings offer new insights into the mechanisms underlying the heterogeneity of asthma.

## AUTHOR CONTRIBUTIONS

All authors were involved in the conception and design of the study. Han‐Kyul Kim, Bermseok Oh, and Tae‐Bum Kim initiated the research project. Han‐Kyul Kim, Ji‐One Kang, Ji Eun Lim, Tae‐Woong Ha, Hae Un Jung, Won Jun Lee, Dong Jun Kim, and Eun Ju Baek analysed the data and prepared figures and tables. Han‐Kyul Kim, Ji‐One Kang, Bermseok Oh, and Tae‐Bum Kim wrote the manuscript, and Ian M. Adcock and Kian Fan Chung contributed to the manuscript writing.

## CONFLICT OF INTEREST STATEMENT

The authors declare no conflicts of interest.

## Supporting information

Supporting Information S1

Supporting Information S2

Supporting Information S3

Supporting Information S4

## Data Availability

The data that support the findings of this study are available from the corresponding author upon reasonable request.
