# Supplementary material for: Genetic differences according to onset age and lung function in asthma: A cluster analysis
Source: Clin Transl Allergy. 2023 Jul 14;13(7):e12282. doi: 10.1002/clt2.12282 (PMC10345724; doi:10.1002/clt2.12282)
Supplement: Supplementary file 1 — Supporting Information S1 [file CLT2-13-e12282-s003.docx]

**Supporting Information**

**Genetic differences according to onset age and lung function in asthma: a cluster analysis**

Han-Kyul Kim^1†^, Ji-One Kang^1†^, Ji Eun Lim^1^, Tae-Woong Ha^1^, Hae Un Jung^2^, Won Jun Lee^1^, Dong Jun Kim^2^, Eun Ju Baek^2,5^, Ian M Adcock^4^, Kian Fan Chung^4^, Tae-Bum Kim^3^*, Bermseok Oh^1,2,5^*

^1^Department of Biochemistry and Molecular Biology, School of Medicine, Kyung Hee University, Seoul 02447, Korea

^2^Department of Biomedical Science, Graduate School, Kyung Hee University, Seoul 02447, Korea

^3^Department of Allergy and Clinical Immunology, Asan Medical Center, University of Ulsan College of Medicine, Seoul 05505, Korea

^4^The National Heart and Lung Institute, Imperial College, London, UK

^5^Mendel, Seoul 02455, Korea

^†^These authors share co-first authors.

*Corresponding authors:

Bermseok Oh

Department of Biochemistry and Molecular Biology, School of Medicine, Kyung Hee University, Seoul 02447, Korea

Phone: +82 2-961-0617

Mobile: +82 10-8140-1534

E-mail: ohbs@khu.ac.kr

Tae-Bum Kim

Department of Allergy and Clinical Immunology, Asan Medical Center, University of Ulsan College of Medicine, Seoul 05505, Korea.

E-mail: tbkim@amc.seoul.kr

**Supplementary Tables**

Supplementary Table S1. Medicines relevant to asthma subjects including UKB id code

Supplementary Table S2. Overview of traits tested for genetic correlation to asthma clusters

Supplementary Table S3. Summary statistics for genome-wide significant signals in total asthma

Supplementary Table S4. Summary statistics for genome-wide significant signals in cluster 1

Supplementary Table S5. Summary statistics for genome-wide significant signals in cluster 2

Supplementary Table S6. Summary statistics for genome-wide significant signals in cluster 3

Supplementary Table S7. Summary statistics for genome-wide significant signals in cluster 4

Supplementary Table S8. Consequence for lead SNPs

Supplementary Table S9. Conditional analysises for 14 independent signals in asthmatic studies

Supplementary Table S10 Similarity between previously reported SNPs and lead SNPs

Supplementary Table S11. Summary of validated lead SNPs

Supplementary Table S12. Lead SNPs validated in the meta-analysis using the unused white set and

moderate-to-severe study

Supplementary Table S13. Summary of cluster lead SNPs validated in the unused British and Irish set

Supplementary Table S14. Cluster lead SNPs validated in the unused British and Irish set

Supplementary Table S15. Overview of traits tested for genetic correlation to asthma clusters

Supplementary Table S16. Summary of gene-based analysis in significant gene-sets in total asthma

Supplementary Table S17. Summary of gene-based analysis in significant gene-sets in cluster 1

Supplementary Table S18. Summary of gene-based analysis in significant gene-sets in cluster 2

Supplementary Table S19. Significant pathways associated with asthma clusters

Supplementary Table S20. Functional consequences on genes of all GWAS siginificant SNPs and lead

SNPs for all studies

Supplementary Table S21. Minimum 15-core chromatin state of all GWAS siginificant SNPs and lead

SNPs for all studies

Supplementary Table S22. Regulome database score of of all GWAS siginificant SNPs and lead SNPs

for all studies

Supplementary Table S23. Results of eQTL analyses for lead SNPs variants with susceptibility to

clusters and total asthma

Supplementary Table S24. Results of eQTL analyses for cluster-specific lead SNPs

Supplementary Table S25. Summary statistics for gene-based analysis from MAGMA

Supplementary Table S26. Full tissue expression profile results from GTEx for asthmatic studies

Supplementary Table S27. Comparison test results of phenotypes between clusters

Supplementary Table S28. Results for lead SNPs using PLINK and SAIGE

Supplementary Table S29. The UK Biobank data field and code

**Supplementary Result**

**Functional annotation of association signals**

We summarised the functional annotations of all genome-wide significant SNPs and all lead SNPs separately in All and clusters **Tables S20, S21, and S22**). Positional information on associated SNPs relevant to the nearest genes (**Table S20**), chromatin state information [1], (**Table S21**) and Regulome DB score information (https://regulomedb.org/regulome-search/) [2] (**Table S22**) are also summarised. Over 80% of lead SNPs from each GWAS were in intergenic and intronic regions, over 89% of lead SNPs from each GWAS belonged to the active state based on information of 15-core chromatin states, and over 62% of lead SNPs showed at least one indication towards transcription regulation based on diverse indexes used for the Roadmap database scores, such as eQTL, TF binding, TF motif, and DNase hypersensitivity.

We also investigated eQTL information on all 163 lead SNPs from available eQTL databases (Methods section). In case no eQTL was found for a lead SNP (sentinel SNP), we used proxy SNPs (*r^2^* > 0.4; within 1 Mb of the region flanking the lead SNP) for the analysis. The results revealed 109 eQTL SNPs (62 sentinel eQTL SNPs and 47 proxy eQTL SNPs) among the 163 lead SNPs, and these 109 eQTL SNPs were significantly associated with the 350 differentially expressed genes in selected tissues or cells such as the blood, lung, spleen, and oesophagus; B cell; T cell; monocyte and platelet (**Table S23**). Among these 109 eQTL SNPs, the genes nearest to the 56 lead SNPs were identical to the eQTL genes (51.4%). Overall, 4 eQTL SNPs were found for 6 early-onset asthma^normalLF^-specific SNPs (corresponding to 15 eQTL genes), 29 eQTL SNPs for 38 early-onset asthma^reducedLF^-specific SNPs (corresponding to 110 eQTL genes), 2 eQTL SNPs for 6 late-onset asthma^normalLF^-specific SNPs (corresponding to 11 eQTL genes) and 1 eQTL SNP for 1 late-onset asthma^reducedLF^-specific SNP (corresponding to two eQTL genes) (**Table S24**). For the five novel loci SNPs, we found rs41302867 as an eQTL for signal sequence receptor 1 (*SSR1*) in the blood (via proxy SNP rs9379084, *r*^2^ = 0.61), with the asthma risk allele (A) being associated with decreased expression of *SSR1*; however, no significant eQTL association was observed for the other four novel locus signals.

**Tissue specificity and gene based** **analysis for asthma clusters**

Tissue specificity and gene analysis of cluster GWAS signals were investigated via a gene-based GWAS, using multi-marker analysis of genoMic annotation (MAGMA) [3]. Gene-based association analyses were performed using GWAS summary statistics obtained in this study via ‘the SNP to Gene analysis programme’ in MAGMA. We obtained 418 significant genes in All, 82 in early-onset asthma^normalLF^, 167 in early-onset asthma^reducedLF^, 26 in late-onset asthma^normalLF^, and 41 in late-onset asthma^reducedLF^ (**Table S25**). The gene-wide significance level was defined as a *P* value of < 2.63×10^-6^ (0.05/19,018 genes), which was depicted as a red line in gene-based Manhattan plots (**Figure S7**), and the number of significant genes is shown on Venn diagrams (**Figure S8**). Fifty-two genes overlapped between early-onset asthma^normalLF^ and early-onset asthma^reducedLF^, indicating that many significant genes in early-onset asthma^normalLF^ were also significant in early-onset asthma^reducedLF^ (63.4%, 52 out of 82 early-onset asthma^normalLF^ genes). However, a large portion of early-onset asthma^reducedLF^ genes were cluster-specific (60.5%, 101 out of 167 genes), and 24 genes among the 101 early-onset asthma^reducedLF^-specific genes were not detected in All. Both late-onset asthma^normalLF^ and late-onset asthma^reducedLF^ had fewer significant genes (26 and 41 genes, respectively) than those in early-onset asthma^normalLF^ and early-onset asthma^reducedLF^, with only a single gene shared between them, and many genes in late-onset asthma^reducedLF^ (29 genes) were not detected in All. The Venn diagram of the cluster GWAS lead SNPs is shown for comparison purposes (**Figure S8C**). The general patterns for gene sharing appeared to be similar to those of genome-wide SNP signals. Again, early-onset asthma^normalLF^ shared most SNPs with early-onset asthma^reducedLF^, and early-onset asthma^reducedLF^ had many early-onset asthma^reducedLF^-specific SNPs. In comparison, late-onset asthma^normalLF^ and late-onset asthma^reducedLF^ largely did not exhibit overlapping or shared SNPs.

To examine the tissue specificity of cluster-significant genes, 54 tissue types from the Genotype-Tissue Expression (GTEx) database [4] were analysed using MAGMA (**Table S26**). early-onset asthma^normalLF^ showed a statistically significant difference between the tissue specificity for the spleen and whole blood (*P* = 4.90×10^‑4^ and *P* = 2.98×10^-4^ in the spleen and whole blood, respectively), and early-onset asthma^reducedLF^ showed significance in the lung, small intestine terminal ileum, and spleen (*P* =4.68×10^-4^, *P* =1.51×10^‑4^ and *P* =4.17×10^-4^ in the lung, small intestine terminal ileum, and spleen, respectively) (**Figure S9**). However, no significant tissue specificities were observed for late-onset asthma^normalLF^ and late-onset asthma^reducedLF^.

**Supplementary Discussion**

**Characteristics of the 5 novel loci with respect to genetic location, nearest or within gene, function, and related diseases**

We have identified, to the best of our knowledge, for the first time, 5 novel loci signals namely rs116351845, rs74696793, rs41302867, rs12889006, and rs833914 for their loci for asthma (Table 3). rs116351845 is localized upstream of *FGF10* (268-Kb apart), a member of the fibroblast growth factor family. FGF10 is implicated in idiopathic pulmonary fibrosis, bronchopulmonary dysplasia, and chronic obstructive pulmonary disease and lung function [5, 6]. Furthermore, rs74696793 is an intron variant of *ERGIC1*, which encodes an endoplasmic reticulum-Golgi intermediate compartment (ERGIC) protein suspected of playing a role in cellular transport between the endoplasmic reticulum and Golgi. rs41302867, an intron variant of *RREB1* that showed an eQTL for *DSP, RIOK1*,*SSR1* and *RREB1* in the blood. The decrease in for *DSP, RIOK1*,*SSR1* and *RREB1* expression in the blood were associated with the risk allele (A). RREB1 functions as a zinc finger transcription factor that binds to RAS-responsive elements of gene promoters [7, 8], and SSR1 functions as a glycosylated endoplasmic reticulum membrane receptor associated with protein translocation across the endoplasmic reticulum membrane [9]. Both *RREB1* and *SSR1* are associated with FEV1/FVC ratio [10] and leucocyte counts [11]. In addition, rs12889006, an intron variant of *ZFP36L1*, exists in the active site of both the minimum chromatin state and common chromatin state. ZFP36L1, which is an early response gene induced by various agonists, such as phorbol ester and polypeptide mitogens, functions as a zinc-finger RNA-binding protein that destabilises several mRNA transcripts by promoting removal of their poly(A) tail [12]. ZFP36L1 reportedly ensures accurate variable-diversity-joining recombination and functional immune cell formation [13, 14]. Lastly, rs833914, an intron variant of *SIPA1L3*, encodes a GTPase-activating protein specific for the GTP-binding protein RAP1 and functions in epithelial cell morphogenesis and in the establishment or maintenance of polarity [15, 16]. *SIPA1L3*, expressed in monocytes and B cells according to ProteomicsDB [17], is reportedly involved in haematopoietic progenitor cell differentiation [18].

**Characteristics of the 9 novel loci with respect to genetic location, nearest or within gene, function, and related diseases**

We identified 9 novel signals from loci associated with asthma: 6:32895973:AT:A (Affymetrix SNP chip ID), rs112563428, rs112119265, rs17302823, rs2735102, rs205002, rs4745723, rs3806155 and rs113457465. The genetic variant of 6:32895973:AT:A is located on chromosome 6 and is 88-Kb apart from a previously reported SNP, rs11964504 (6:33015971, *r^2^* = 0.002), which was identified via a nonatopic asthma GWAS [19] and located between *HLA-DPA1* and *HLA-DOA*. The SNP rs112563428, an intron variant of *UHRF1BP1* (UHRF1 binding protein 1), was 344-Kb apart from the reported SNP rs28522747 (6:35166952, *r^2^* = 0.014) [20], which was located between *SCUBE3* (signal peptide, CUB domain, and EGF like domain containing 3) and *TCP11* (t-complex 11). The SNP rs112563428 has a proxy SNP, rs3800461 (*r^2^* = 0.75), which yields an eQTL signal corresponding to inflammation and lipid regulator with UBA-Like and NBR1-like domains that functions as a negative regulator of innate antiviral response by blocking IRF3-dependent cytokine production such as IFNA, IFNB, and TNF [21]. The SNP rs112119265, an intron variant of *CARD11* (caspase recruitment domain family member 11), was 87-Kb apart from the reported SNP rs73033536 (7:3110249, *r^2^* = 0.014), which was identified in both All asthma and non-atopic asthma GWASs [19, 22] and located within the unknown gene LOC105375130. The CARD domain of this protein specifically interacts with BCL10 (BCL10 immune signalling adaptor), a positive regulator of cell apoptosis and NF-κB activation. *CARD11* mutation causes B-cell expansion with NF-κB and T-cell anergy [23]. The SNP rs17302823, an intergenic variant localised between *LOC401312* and interleukin 6 (*IL6*), was 56-Kb apart from the reported SNP rs34880821 (7:22735831, *r^2^* = 0.081), which was identified as being involved in childhood asthma [24] and located between *IL6* and MT-CYB pseudogene 42. IL6, a key cytokine involved in inflammation as well as B-cell maturation [25], drives type 2 inflammation [26]. The SNP rs2735102, an intron variant of *HLA-A*, was 16-Kb apart from the reported SNP rs2517690 (6:29961296, *r^2^* = 0.002), which was identified in atopic asthma [19], and located between MHC class I polypeptide-related sequence D (pseudogene) and *HLA-W*. The SNP rs205002, an intergenic variant between activating transcription factor 6 beta (*ATF6B*) and *PRRT1*, was 42-Kb apart from the reported SNP rs204993 (6:32187804, *r^2^* = 0.013) [27] located within the PBX homeobox 2 gene. Genetic variants of *ATF6B* are associated with chronic obstructive pulmonary disease and ever-smoker interaction (main effect) [28]. In addition, rs41284471 has a proxy SNP, rs2255088 (*r^2^* = 0.56), yielding an eQTL corresponding to the protein kinase C theta (*PRKCQ*) gene in the blood (**Table S23**). PRKCQ, a serine- and threonine-specific protein kinase, is known for T-cell activation [29] and for possibly linking T-cell receptor signalling complex to the activation of transcription factors such as NF-κB and AP-1 [30]. Diseases associated with *PRKCQ* include Crohn’s disease and inflammatory bowel disease 1 [31]. The SNP rs4745723, an intron variant of *CAMK2G*, was 65-Kb apart from the reported SNP rs1134777 (10:73778893, *r^2^* = 0.059) [22], located within the fucosyltransferase 11 gene. CAMK2G is a gamma subunit of the four subunits of Ca^+2^/calmodulin-dependent protein kinase II and functions autonomously after Ca^2+^/calmodulin-binding and autophosphorylation, involved in sarcoplasmic reticulum Ca^2+^ transport in skeletal muscle, and also functions in dendritic spine and synapse formation and neuronal plasticity [32, 33], however, this protein is seldom associated with immune response.

A proxy SNP, rs2306327 (r^2^ = 0.61) of rs4745723, was found to have an eQTL signal corresponding to N-deacetylase and N-sulfotransferase 2 (*NDST2*) in the blood. Studies have shown that NDST2^−/−^ mice have a deficiency in mast cell protease storage due to the absence of a fully sulphated heparin structure[34, 35]. The role of mast cell proteases in asthma was revealed by evaluating mouse allergic airway inflammatory knockout models [36].

The SNP rs3806155, an intron variant of both TSBP1 and BTNL2 antisense RNA 1 (*TSBP1-AS1*) and *BTNL2*, was 15-Kb apart from the reported SNP rs3117098 (6:32390736, *r^2^* = 0.006) [22], located within *TSBP1-AS1*. BTNL2 is a major histocompatibility complex, class II-associated, type I transmembrane protein that belongs to the butyrophilin-like B7 family of immunoregulators and is thought to be involved in immune surveillance, serving as a negative T-cell regulator by decreasing T-cell proliferation and cytokine release [37]. Naturally occurring mutations in this gene are associated with sarcoidosis, rheumatoid arthritis, ulcerative colitis, inflammatory bowel disease, myositis, type 1 diabetes, systemic lupus erythematosus, acute coronary syndrome, and prostate cancer [38-40]. The SNP rs113457465, an intergenic variant between *HLA-DQB1* and *HLA-DQA2*, was 39-Kb apart from the reported SNP rs17843577 (6:32647733, *r^2^* = 0.00) [41] located between *HLA-DQA1* and *HLA-DQB1*.

**Characteristics of the 8 early-onset asthma^reducedLF^-specific lead SNPs related to signalling for cell activation**

Investigation of eQTL genes for early-onset asthma^reducedLF^-specific lead SNPs revealed genes related to signalling for cell activation, including *TNFSF4* (rs4090390), *ITPKB* (rs3768410), *PLCL1* (rs1318867), *BACH2* (rs72928038), *THEMIS* (rs41285280), *ZBTB10* (rs6473228), *PRKCQ-AS* (rs41284471), and *UBAC2* (rs9517640). The rs4090390 is an eQTL for *TNFSF4*, which is known for T-cell activation, and the minor allele of this SNP (‘A’ nucleotide) reduces the expression of *TNFSF4*, resulting in a decreased incidence of asthma (**Table S5** and **S24**). Moreover, rs3768410 is an eQTL for *ITPKB*, which negatively regulates signal transduction by phosphorylating the second messenger, inositol-1,4,5 triphosphate, leading to the inactivation of the signal, and the minor allele of this SNP (‘T’ nucleotide) increases the expression of *ITPKB*, resulting in a decreased incidence of asthma. In addition, rs1318867 has a proxy SNP, rs771016 (*r^2^* = 0.65), with an eQTL for *PLCL1*, which is involved in inositol phospholipid-based intracellular signalling, and the minor allele of this proxy SNP (‘C’ nucleotide) increases *PLCL1* expression, resulting in an increased incidence of asthma. Furthermore, rs72928038 is an eQTL for *BACH2*, which functions as a transcriptional regulator for leucocyte proliferation involved in NF-κB signalling, and the minor allele of this SNP (‘A’ nucleotide) reduces *BACH2* expression, resulting in a decreased incidence of asthma. BACH2 is a transcription factor specifically expressed in B and T cells and regulates the lineage and maturation of these cells [42]. Further, rs41285280 is an eQTL for *THEMIS*, which regulates T-cell receptor signalling in the thymus resulting in T-cell selection and maturation, and the minor allele of this SNP (‘T’ nucleotide) increases the expression of *THEMIS*, resulting in an increased incidence of asthma. Mice with the loss of *THEMIS* show a decreased number of single positive T cells in the blood [43]. The number rs6473228 represents an eQTL for *ZBTB10*, a transcriptional regulator, and the minor allele of this SNP (‘G’ nucleotide) increases *ZBTB10* expression, thereby increasing the incidence of asthma. An eQTL for *PRKCQ-AS* is represented by rs41284471, which targets PRKCQ, a protein known to link T-cell receptor signalling to transcriptional activation described above, and the minor allele of this SNP (‘A’ nucleotide) reduces *PRKCQ-AS* expression and consequently increases the incidence of asthma. The eQTL for *UBAC2*, a negative regulator of Wnt signalling in lymphocytes, is represented by rs9517640, and the minor allele of this SNP (‘A’ nucleotide) increases *UBAC2* expression, resulting in a decreased incidence of asthma. These results suggest that increased cell activation signalling increases the incidence of early-onset asthma^reducedLF^ asthma, whereas decreased signalling due to activation of negative regulators decreases the incidence of early-onset asthma^reducedLF^ asthma.

**Characteristics of early-onset asthma^reducedLF^-specific lead SNPs related to pathogen recognition, cytokine receptors, and T-cell differentiation**

In addition to the signalling for cell activation, the investigation of eQTL genes for early-onset asthma^reducedLF^-specific lead SNPs revealed gene regulating pathogen recognition, such as *TLR1, TLR6, TLR10* (rs11728049, rs2890664), and nucleotide binding oligomerisation domain containing 2 (NOD2; rs12324931), as well as cytokine receptors, such as C-C motif chemokine receptor 4 (*CCR4*; rs35570272) and *CXCR5* (rs12365699) and T-cell differentiation, such as IL4R (rs3785356), *RORA* (rs34986765), and *RORC* (rs3828058), were identified.

Related to pathogen recognition, rs11728049, an intergenic variant localised between *LOC105374412* and *TLR10*, yielded an eQTL corresponding to *TLR1* and *TLR6* in the blood, T cells, and oesophageal mucosa, each of which plays a fundamental role in pathogen recognition and activation of innate immunity [44]. TLRs recognise pathogen-associated molecular patterns expressed on infectious agents and mediate the production of cytokines necessary for the development of effective immunity. The minor allele of this SNP (‘G’ nucleotide) reduces the expression of *TLR1* and *TLR6*, resulting in a decreased incidence of asthma (OR = 0.84). In addition, rs2890664, a 3′ UTR variant of *TLR6*, has proxy SNPs that yield eQTLs corresponding to TLRs (rs3775073, *r^2^* = 0.49, *TLR6* and rs4691002, *r^2^* = 0.41 *TLR1*) in the blood. *TLR1* and *TLR6* form a heterodimer with *TLR2* to play a fundamental role in pathogen recognition and activation of innate immunity [45]. In a mouse model, TLR2/TLR6 functions as a key receptor that controls respiratory syncytial virus [46], which is closely related to the development of childhood asthma [47]. Both rs11728049 and rs2890664 showed potential associations with early-onset asthma^normalLF^ (*P* = 3.56 × 10^-7^ and 2.96 × 10^-6^, respectively). Furthermore, one more lead SNP rs140633479 showed an eQTL signal corresponding to *TLR1* and *TLR6*, demonstrating early-onset cluster specificity (early-onset asthma^normalLF^ and early-onset asthma^reducedLF^; **Table S8**). The minor allele of this SNP (‘AAATAAAT’ nucleotide) reduces the expression of *TLR1* and *TLR6*, resulting in the decreased incidence of asthma (OR = 0.86). Based on these results, we speculated that pathogen recognition by *TLR1* and *TLR6*, probably via the heterodimer formed with TLR2, may be important for inducing and exacerbating early-onset asthma. Furthermore, rs12324931, an intron variant of CYLD lysine 63 deubiquitinase, yields an eQTL signal corresponding to *NOD2*, which encodes an intracellular pattern recognition receptor that plays an important role in recognising pathogens and initiating immune response [48]. Therefore, both intracellular and extracellular recognition of pathogens, followed by an immune response, may be involved in inducing early-onset asthma.

In terms of cytokine receptors, an intronic variant, rs35570272, located in the galactosidase beta 1 gene, showed an eQTL signal corresponding to CCR4 in the blood. CCR4 plays a crucial role in the recruitment of T cells in the asthmatic airway [49]. Inhibiting CCR4 effectively suppresses the secretion of Th2 cytokines, which prevents airway eosinophilia and hyper-responsiveness [50].

An eQTL associated with rs12365699, located 11-Kb upstream of *CXCR5*, corresponded to *CXCR5* in the blood and showed a high Regulome DB score (**Table S22**). CXCR5, a receptor for CXCL13 ligand, is expressed on B cells and CD4^+^ T cells and is related to acute asthma patients with a positive correlation with total IgE [51].

Regarding T-cell differentiation, rs3785356, an intron variant of *IL4R*, yields an eQTL corresponding to *IL4R* in the blood and lungs as well as monocytes, which encodes the alpha chain of the interleukin-4 receptor, a type I transmembrane protein that can bind interleukin 4 and interleukin 13 to regulate IgE production [52]. Moreover, IL4R can bind to interleukin 4 to promote Th2 cell differentiation [53]. The Th2 pathway is involved in the exacerbation of childhood-onset asthma [54]. The SNP rs3828058, an intron variant of *RORC*, yields an eQTL signal corresponding to *RORC*, and rs34986765, an intron variant of *RORA*, yields an eQTL signal corresponding to *RORA*. RORC plays a critical role as a transcription factor for Th17 differentiation, termed as proinflammatory activity [55]. In young patients with asthma, the ratio of *RORC* expression to *FOXP3*, a Treg marker, was increased, compounding the role of *RORC* in the incidence of early-onset asthma [44]. IL‑2 inhibits Th17 cell development by downregulating the expression of Th17 cell-associated *RORC* [56]. In contrast, *RORA* may play an essential role in the development of type 2 innate lymphoid cells (ILC2s), supported by the lack of ILC2s cells in RORA-deficient mice [57].

**Supplementary Figures**


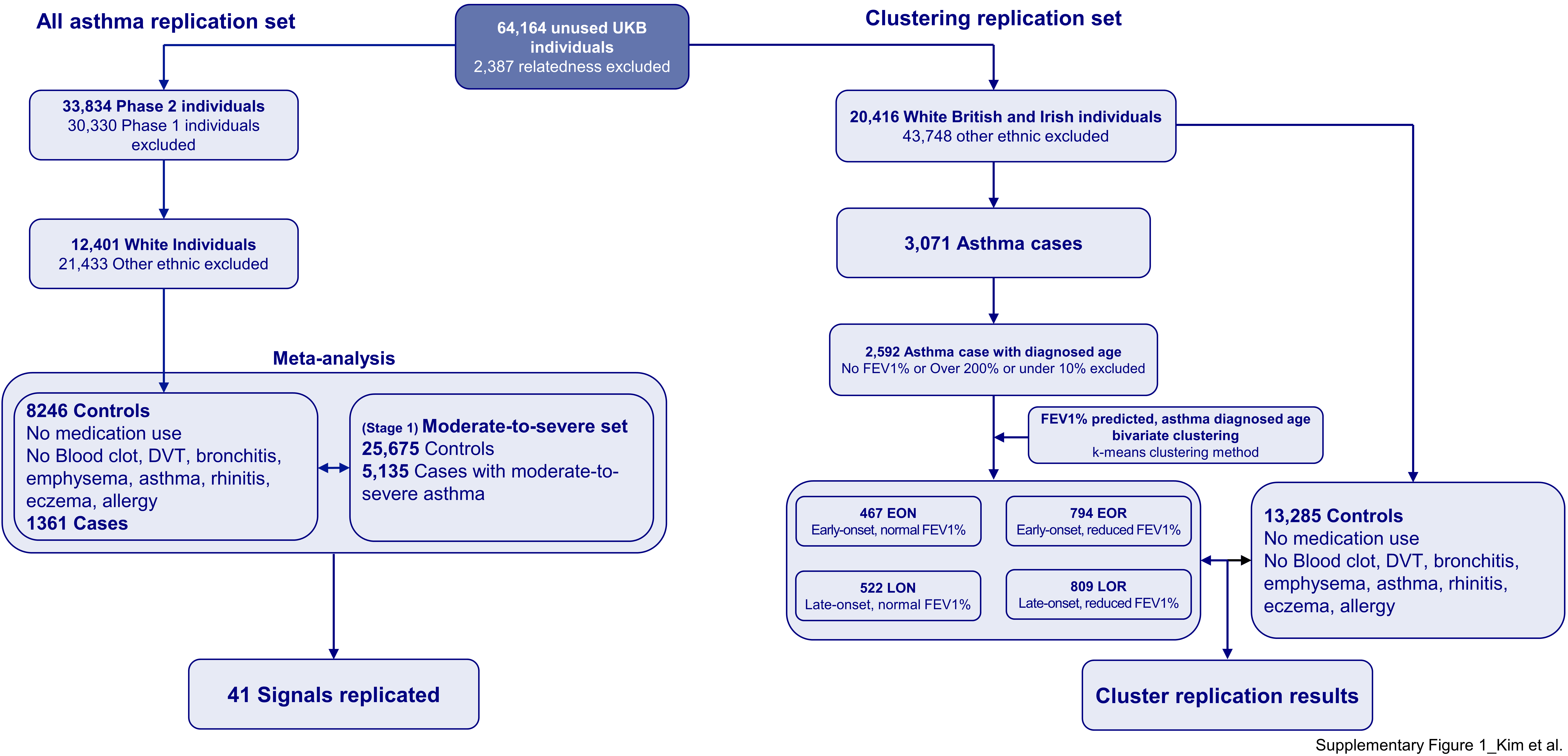


**Supplementary Figure S1: Diagram of the replication study design**


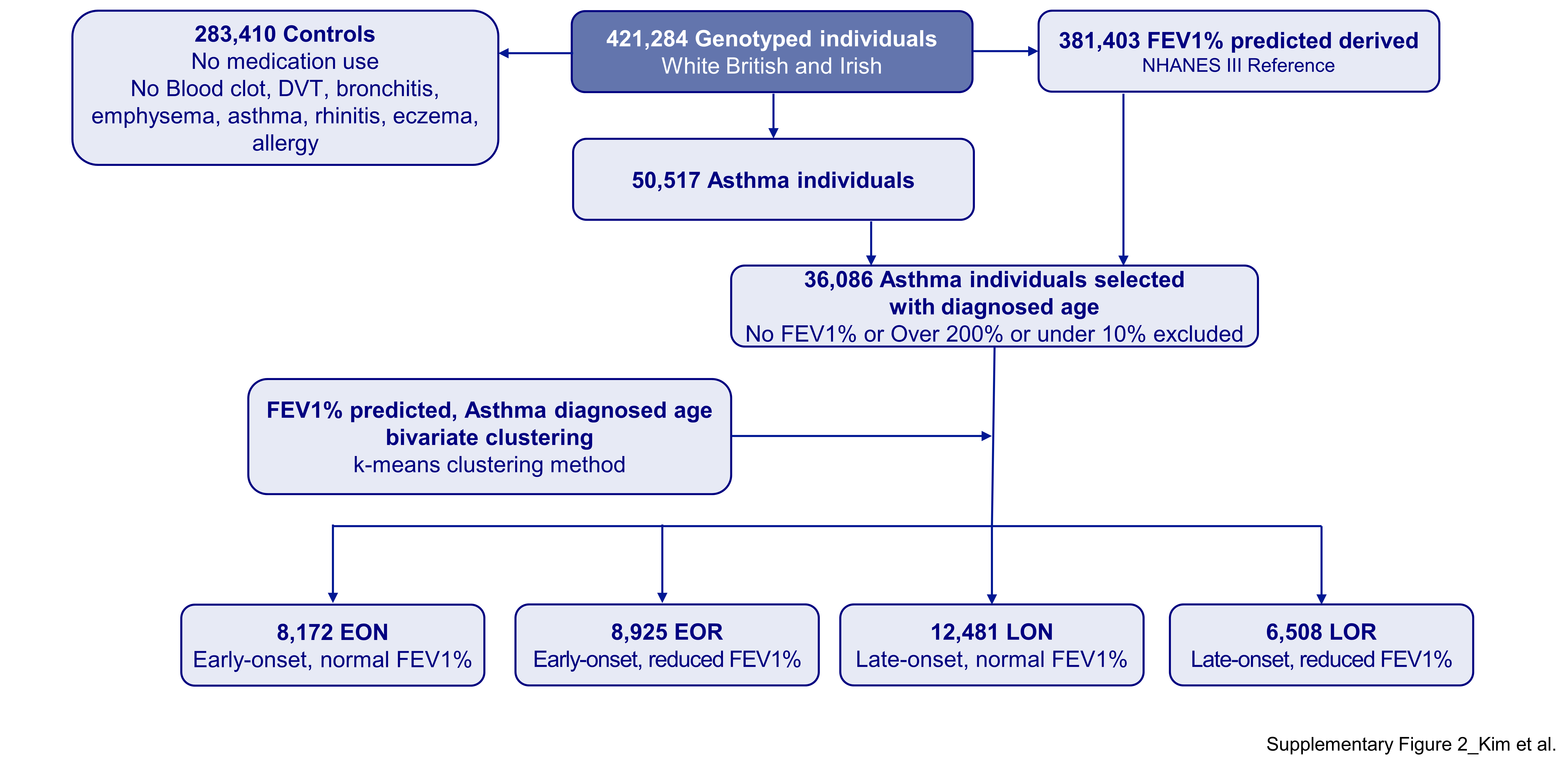


**Supplementary Figure S2: Diagram of the overall clustering design.**

**
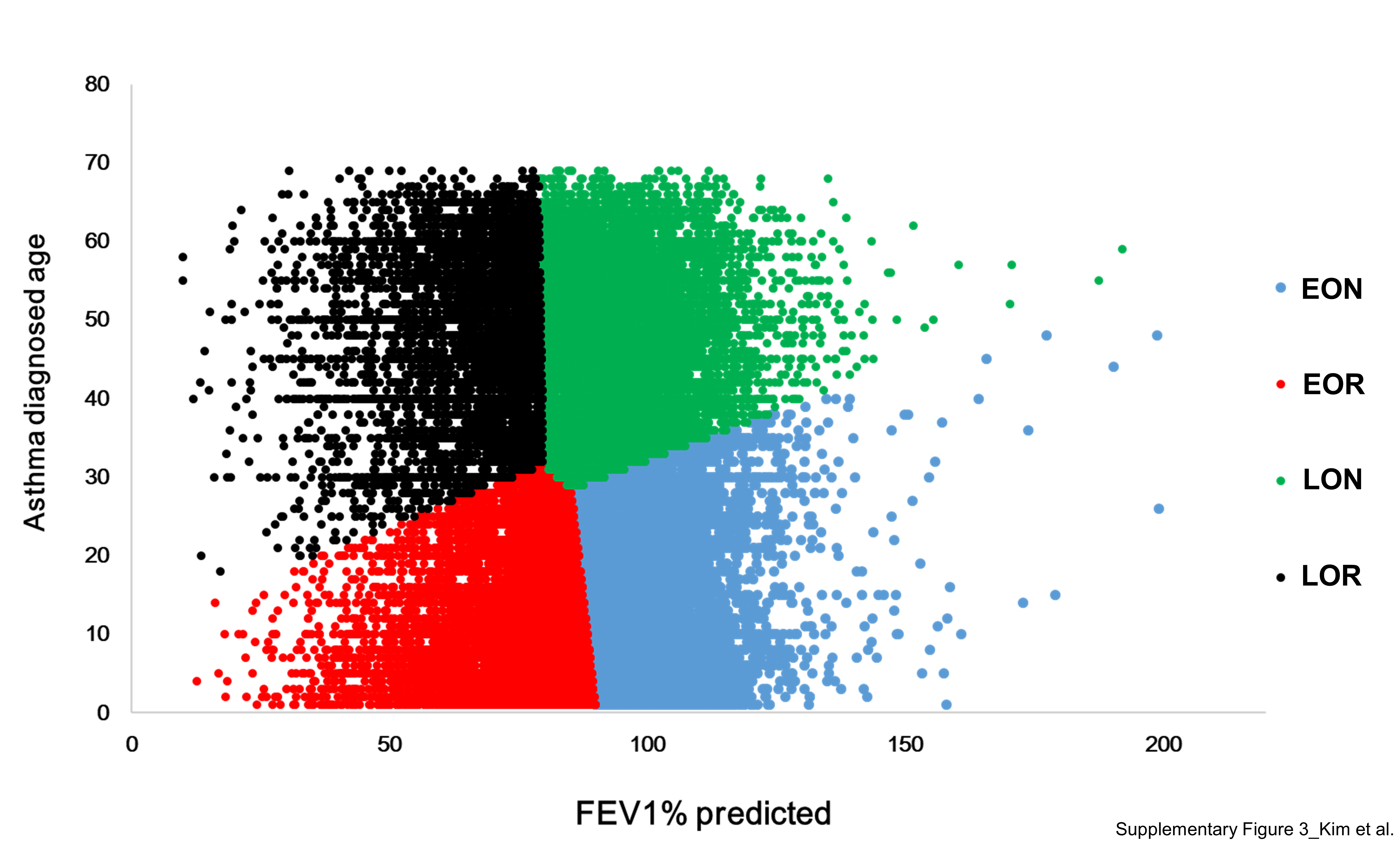
**

**Supplementary Figure S3: Scatter plot of patients with asthma for two clustering variables.** Scatter plot of patients with asthma for the variables of onset age and FEV1% predicted used in the clustering analysis was generated using SPSS. Most individuals in early-onset asthma^normalLF^ (blue) and late-onset asthma^normalLF^ (green) had 89% or higher and 80% or higher FEV1% predicted, respectively, and the onset age of individuals in both early-onset asthma^normalLF^ and early-onset asthma^reducedLF^ (red) was mostly less than 25 years old.

**
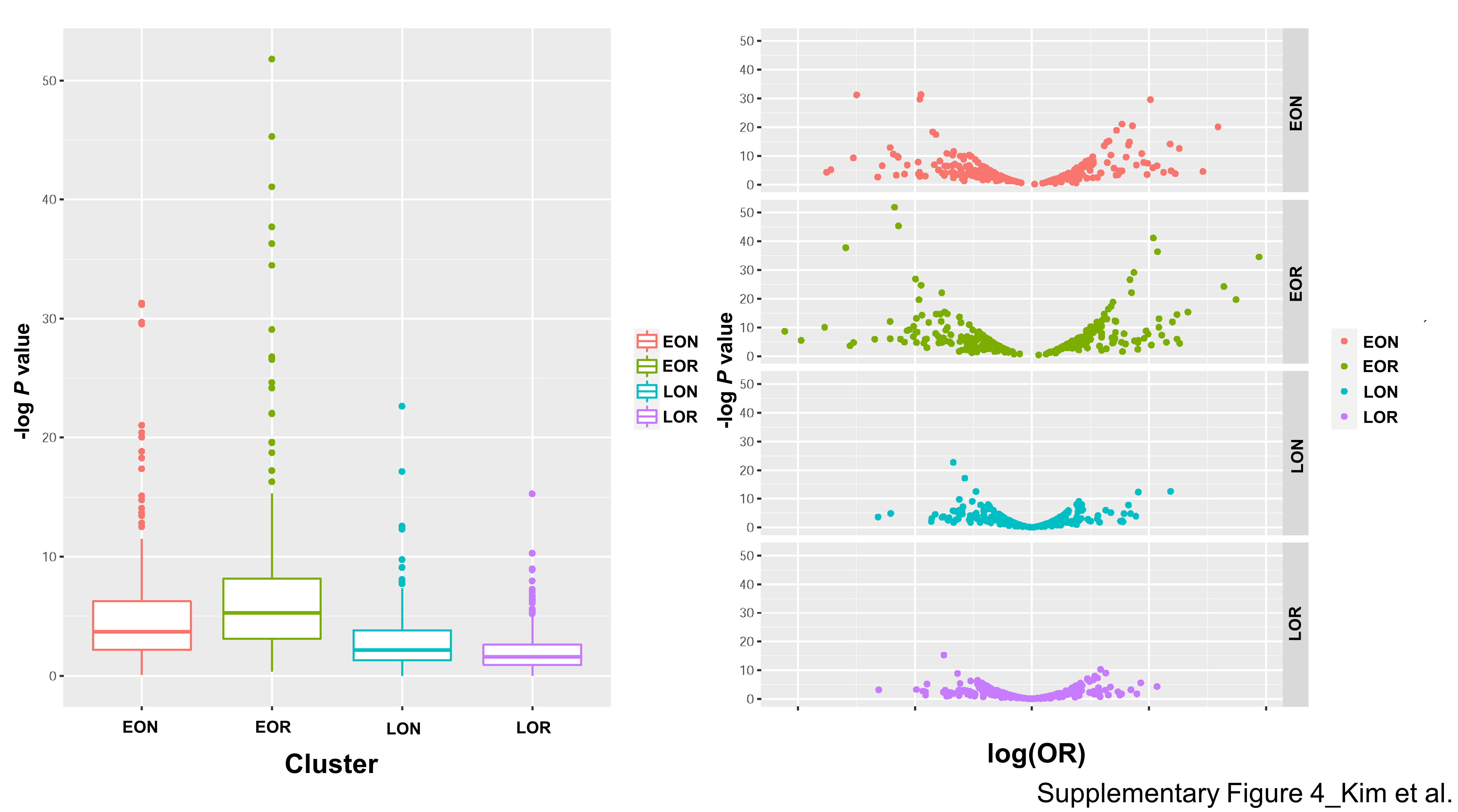
**

**Supplementary Figure S4: Box plot and volcano plot for P values and odd ratio.** A box plot of -log P values for 163 lead SNPs with the defined clusters. Outliers are indicated with small circles. Volcano plot of GWAS results from each cluster showing the -log P value and odd ratio. The x axis shows the odd ratio of lead SNPs, while the y axis shows the -log P value for the associations per SNPs.

**
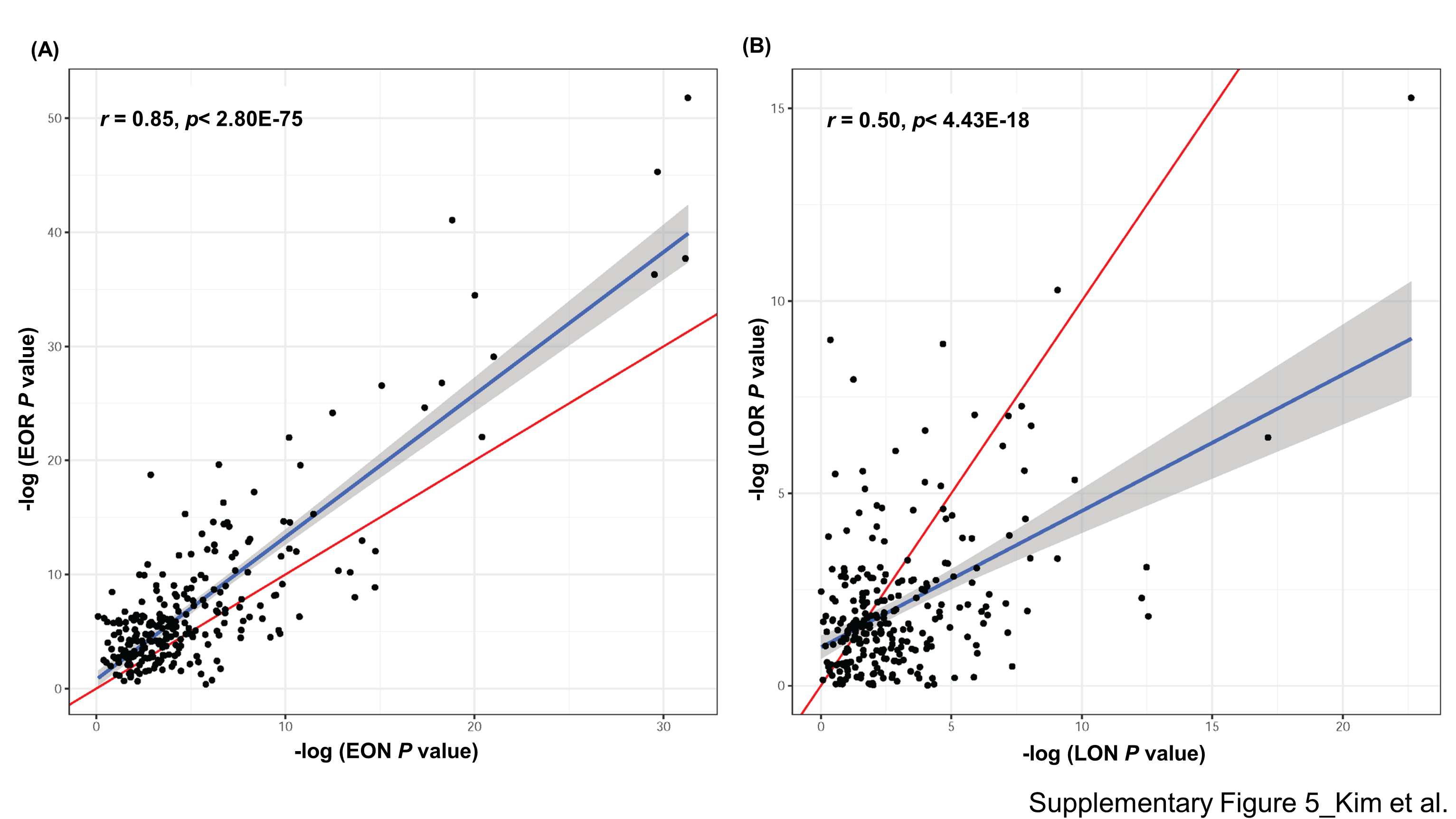
**

**Supplementary Figure S5: Scatter plot for P values.** Scatter plots of -log P values for 163 lead SNPs in the GWAS of early-onset asthma subgroups (early-onset asthma^normalLF^ and early-onset asthma^reducedLF^) (A) and late-onset subgroups (late-onset asthma^normalLF^ and late-onset asthma^reducedLF^) (B) were generated for each cluster, with trend lines shown in blue and the line y = x highlighted in red. The correlation coefficient (r) and P-values were estimated using the Pearson method. The red line represents the model fitted for two groups with hypothetically identical P values (r = 1), while the blue line represents the model fitted for two groups with true P values.

**References**

1. Ernst J, Kellis M: **Chromatin-state discovery and genome annotation with ChromHMM.** *Nat Protoc* 2017, **12:**2478-2492.

2. Boyle AP, Hong EL, Hariharan M, Cheng Y, Schaub MA, Kasowski M, Karczewski KJ, Park J, Hitz BC, Weng S, et al: **Annotation of functional variation in personal genomes using RegulomeDB.** *Genome Res* 2012, **22:**1790-1797.

3. de Leeuw CA, Mooij JM, Heskes T, Posthuma D: **MAGMA: generalized gene-set analysis of GWAS data.** *PLoS Comput Biol* 2015, **11:**e1004219.

4. **Human genomics. The Genotype-Tissue Expression (GTEx) pilot analysis: multitissue gene regulation in humans.** *Science* 2015, **348:**648-660.

5. Yuan T, Volckaert T, Chanda D, Thannickal VJ, De Langhe SP: **Fgf10 Signaling in Lung Development, Homeostasis, Disease, and Repair After Injury.** *Front Genet* 2018, **9:**418.

6. Jackson VE, Latourelle JC, Wain LV, Smith AV, Grove ML, Bartz TM, Obeidat M, Province MA, Gao W, Qaiser B, et al: **Meta-analysis of exome array data identifies six novel genetic loci for lung function.** *Wellcome Open Res* 2018, **3:**4.

7. Fujimoto-Nishiyama A, Ishii S, Matsuda S, Inoue J, Yamamoto T: **A novel zinc finger protein, Finb, is a transcriptional activator and localized in nuclear bodies.** *Gene* 1997, **195:**267-275.

8. Date S, Nibu Y, Yanai K, Hirata J, Yagami K, Fukamizu A: **Finb, a multiple zinc finger protein, represses transcription of the human angiotensinogen gene.** *Int J Mol Med* 2004, **13:**637-642.

9. Pfeffer S, Dudek J, Schaffer M, Ng BG, Albert S, Plitzko JM, Baumeister W, Zimmermann R, Freeze HH, Engel BD, Forster F: **Dissecting the molecular organization of the translocon-associated protein complex.** *Nat Commun* 2017, **8:**14516.

10. Kichaev G, Bhatia G, Loh PR, Gazal S, Burch K, Freund MK, Schoech A, Pasaniuc B, Price AL: **Leveraging Polygenic Functional Enrichment to Improve GWAS Power.** *Am J Hum Genet* 2019, **104:**65-75.

11. Astle WJ, Elding H, Jiang T, Allen D, Ruklisa D, Mann AL, Mead D, Bouman H, Riveros-Mckay F, Kostadima MA, et al: **The Allelic Landscape of Human Blood Cell Trait Variation and Links to Common Complex Disease.** *Cell* 2016, **167:**1415-1429 e1419.

12. Lai WS, Kennington EA, Blackshear PJ: **Tristetraprolin and its family members can promote the cell-free deadenylation of AU-rich element-containing mRNAs by poly(A) ribonuclease.** *Mol Cell Biol* 2003, **23:**3798-3812.

13. Holgate ST: **Mechanisms of asthma and implications for its prevention and treatment: a personal journey.** *Allergy Asthma Immunol Res* 2013, **5:**343-347.

14. Galloway A, Saveliev A, Lukasiak S, Hodson DJ, Bolland D, Balmanno K, Ahlfors H, Monzon-Casanova E, Mannurita SC, Bell LS, et al: **RNA-binding proteins ZFP36L1 and ZFP36L2 promote cell quiescence.** *Science* 2016, **352:**453-459.

15. Kooistra MR, Dube N, Bos JL: **Rap1: a key regulator in cell-cell junction formation.** *J Cell Sci* 2007, **120:**17-22.

16. Greenlees R, Mihelec M, Yousoof S, Speidel D, Wu SK, Rinkwitz S, Prokudin I, Perveen R, Cheng A, Ma A, et al: **Mutations in SIPA1L3 cause eye defects through disruption of cell polarity and cytoskeleton organization.** *Hum Mol Genet* 2015, **24:**5789-5804.

17. Fishilevich S, Zimmerman S, Kohn A, Iny Stein T, Olender T, Kolker E, Safran M, Lancet D: **Genic insights from integrated human proteomics in GeneCards.** *Database (Oxford)* 2016, **2016**.

18. **Gene Ontology Consortium: going forward.** *Nucleic Acids Res* 2015, **43:**D1049-1056.

19. Zhu Z, Guo Y, Shi H, Liu CL, Panganiban RA, Chung W, O'Connor LJ, Himes BE, Gazal S, Hasegawa K, et al: **Shared genetic and experimental links between obesity-related traits and asthma subtypes in UK Biobank.** *J Allergy Clin Immunol* 2020, **145:**537-549.

20. Ferreira MAR, Mathur R, Vonk JM, Szwajda A, Brumpton B, Granell R, Brew BK, Ullemar V, Lu Y, Jiang Y, et al: **Genetic Architectures of Childhood- and Adult-Onset Asthma Are Partly Distinct.** *Am J Hum Genet* 2019, **104:**665-684.

21. Ambrose RL, Liu YC, Adams TE, Bean AGD, Stewart CR: **C6orf106 is a novel inhibitor of the interferon-regulatory factor 3-dependent innate antiviral response.** *J Biol Chem* 2018, **293:**10561-10573.

22. Han Y, Jia Q, Jahani PS, Hurrell BP, Pan C, Huang P, Gukasyan J, Woodward NC, Eskin E, Gilliland FD, et al: **Genome-wide analysis highlights contribution of immune system pathways to the genetic architecture of asthma.** *Nat Commun* 2020, **11:**1776.

23. Gupta M, Aluri J, Desai M, Lokeshwar M, Taur P, Lenardo M, Bergerson J, Dalvi A, Mhatre S, Kulkarni M, et al: **Clinical, Immunological, and Molecular Findings in Four Cases of B Cell Expansion With NF-kappaB and T Cell Anergy Disease for the First Time From India.** *Front Immunol* 2018, **9:**1049.

24. Pividori M, Schoettler N, Nicolae DL, Ober C, Im HK: **Shared and distinct genetic risk factors for childhood-onset and adult-onset asthma: genome-wide and transcriptome-wide studies.** *Lancet Respir Med* 2019, **7:**509-522.

25. Muraguchi A, Hirano T, Tang B, Matsuda T, Horii Y, Nakajima K, Kishimoto T: **The essential role of B cell stimulatory factor 2 (BSF-2/IL-6) for the terminal differentiation of B cells.** *J Exp Med* 1988, **167:**332-344.

26. Rincon M, Irvin CG: **Role of IL-6 in asthma and other inflammatory pulmonary diseases.** *Int J Biol Sci* 2012, **8:**1281-1290.

27. Hirota T, Takahashi A, Kubo M, Tsunoda T, Tomita K, Doi S, Fujita K, Miyatake A, Enomoto T, Miyagawa T, et al: **Genome-wide association study identifies three new susceptibility loci for adult asthma in the Japanese population.** *Nat Genet* 2011, **43:**893-896.

28. Kim W, Prokopenko D, Sakornsakolpat P, Hobbs BD, Lutz SM, Hokanson JE, Wain LV, Melbourne CA, Shrine N, Tobin MD, et al: **Genome-Wide Gene-by-Smoking Interaction Study of Chronic Obstructive Pulmonary Disease.** *Am J Epidemiol* 2021, **190:**875-885.

29. Altman A, Isakov N, Baier G: **Protein kinase Ctheta: a new essential superstar on the T-cell stage.** *Immunol Today* 2000, **21:**567-573.

30. Gruber T, Pfeifhofer-Obermair C, Baier G: **PKCtheta is necessary for efficient activation of NFkappaB, NFAT, and AP-1 during positive selection of thymocytes.** *Immunol Lett* 2010, **132:**6-11.

31. Yang L, Yan Y: **Protein kinases are potential targets to treat inflammatory bowel disease.** *World J Gastrointest Pharmacol Ther* 2014, **5:**209-217.

32. Rose AJ, Kiens B, Richter EA: **Ca2+-calmodulin-dependent protein kinase expression and signalling in skeletal muscle during exercise.** *J Physiol* 2006, **574:**889-903.

33. Chin ER: **Role of Ca2+/calmodulin-dependent kinases in skeletal muscle plasticity.** *J Appl Physiol (1985)* 2005, **99:**414-423.

34. Forsberg E, Pejler G, Ringvall M, Lunderius C, Tomasini-Johansson B, Kusche-Gullberg M, Eriksson I, Ledin J, Hellman L, Kjellen L: **Abnormal mast cells in mice deficient in a heparin-synthesizing enzyme.** *Nature* 1999, **400:**773-776.

35. Humphries DE, Wong GW, Friend DS, Gurish MF, Qiu WT, Huang C, Sharpe AH, Stevens RL: **Heparin is essential for the storage of specific granule proteases in mast cells.** *Nature* 1999, **400:**769-772.

36. Pejler G: **The emerging role of mast cell proteases in asthma.** *Eur Respir J* 2019, **54**.

37. Nguyen T, Liu XK, Zhang Y, Dong C: **BTNL2, a butyrophilin-like molecule that functions to inhibit T cell activation.** *J Immunol* 2006, **176:**7354-7360.

38. Fitzgerald LM, Kumar A, Boyle EA, Zhang Y, McIntosh LM, Kolb S, Stott-Miller M, Smith T, Karyadi DM, Ostrander EA, et al: **Germline missense variants in the BTNL2 gene are associated with prostate cancer susceptibility.** *Cancer Epidemiol Biomarkers Prev* 2013, **22:**1520-1528.

39. Hom G, Graham RR, Modrek B, Taylor KE, Ortmann W, Garnier S, Lee AT, Chung SA, Ferreira RC, Pant PV, et al: **Association of systemic lupus erythematosus with C8orf13-BLK and ITGAM-ITGAX.** *N Engl J Med* 2008, **358:**900-909.

40. Arnett HA, Escobar SS, Gonzalez-Suarez E, Budelsky AL, Steffen LA, Boiani N, Zhang M, Siu G, Brewer AW, Viney JL: **BTNL2, a butyrophilin/B7-like molecule, is a negative costimulatory molecule modulated in intestinal inflammation.** *J Immunol* 2007, **178:**1523-1533.

41. Johansson A, Rask-Andersen M, Karlsson T, Ek WE: **Genome-wide association analysis of 350 000 Caucasians from the UK Biobank identifies novel loci for asthma, hay fever and eczema.** *Hum Mol Genet* 2019, **28:**4022-4041.

42. Sidwell T, Kallies A: **Bach2 is required for B cell and T cell memory differentiation.** *Nat Immunol* 2016, **17:**744-745.

43. Fu G, Vallee S, Rybakin V, McGuire MV, Ampudia J, Brockmeyer C, Salek M, Fallen PR, Hoerter JA, Munshi A, et al: **Themis controls thymocyte selection through regulation of T cell antigen receptor-mediated signaling.** *Nat Immunol* 2009, **10:**848-856.

44. Kawasaki T, Kawai T: **Toll-like receptor signaling pathways.** *Front Immunol* 2014, **5:**461.

45. Oliveira-Nascimento L, Massari P, Wetzler LM: **The Role of TLR2 in Infection and Immunity.** *Front Immunol* 2012, **3:**79.

46. Murawski MR, Bowen GN, Cerny AM, Anderson LJ, Haynes LM, Tripp RA, Kurt-Jones EA, Finberg RW: **Respiratory syncytial virus activates innate immunity through Toll-like receptor 2.** *J Virol* 2009, **83:**1492-1500.

47. Perez-Yarza EG, Moreno A, Lazaro P, Mejias A, Ramilo O: **The association between respiratory syncytial virus infection and the development of childhood asthma: a systematic review of the literature.** *Pediatr Infect Dis J* 2007, **26:**733-739.

48. Negroni A, Pierdomenico M, Cucchiara S, Stronati L: **NOD2 and inflammation: current insights.** *J Inflamm Res* 2018, **11:**49-60.

49. Vijayanand P, Durkin K, Hartmann G, Morjaria J, Seumois G, Staples KJ, Hall D, Bessant C, Bartholomew M, Howarth PH, et al: **Chemokine receptor 4 plays a key role in T cell recruitment into the airways of asthmatic patients.** *J Immunol* 2010, **184:**4568-4574.

50. Perros F, Hoogsteden HC, Coyle AJ, Lambrecht BN, Hammad H: **Blockade of CCR4 in a humanized model of asthma reveals a critical role for DC-derived CCL17 and CCL22 in attracting Th2 cells and inducing airway inflammation.** *Allergy* 2009, **64:**995-1002.

51. Gong F, Zhu HY, Zhu J, Dong QJ, Huang X, Jiang DJ: **Circulating CXCR5(+)CD4(+) T cells participate in the IgE accumulation in allergic asthma.** *Immunol Lett* 2018, **197:**9-14.

52. Junttila IS: **Tuning the Cytokine Responses: An Update on Interleukin (IL)-4 and IL-13 Receptor Complexes.** *Front Immunol* 2018, **9:**888.

53. Zhu J: **T helper 2 (Th2) cell differentiation, type 2 innate lymphoid cell (ILC2) development and regulation of interleukin-4 (IL-4) and IL-13 production.** *Cytokine* 2015, **75:**14-24.

54. Kuo CS, Pavlidis S, Loza M, Baribaud F, Rowe A, Pandis I, Sousa A, Corfield J, Djukanovic R, Lutter R, et al: **T-helper cell type 2 (Th2) and non-Th2 molecular phenotypes of asthma using sputum transcriptomics in U-BIOPRED.** *Eur Respir J* 2017, **49**.

55. Capone A, Volpe E: **Transcriptional Regulators of T Helper 17 Cell Differentiation in Health and Autoimmune Diseases.** *Front Immunol* 2020, **11:**348.

56. Kryczek I, Wei S, Vatan L, Escara-Wilke J, Szeliga W, Keller ET, Zou W: **Cutting edge: opposite effects of IL-1 and IL-2 on the regulation of IL-17+ T cell pool IL-1 subverts IL-2-mediated suppression.** *J Immunol* 2007, **179:**1423-1426.

57. Halim TY, MacLaren A, Romanish MT, Gold MJ, McNagny KM, Takei F: **Retinoic-acid-receptor-related orphan nuclear receptor alpha is required for natural helper cell development and allergic inflammation.** *Immunity* 2012, **37:**463-474.
