# Supplementary material for: Genetic differences according to onset age and lung function in asthma: A cluster analysis
Source: Clin Transl Allergy. 2023 Jul 14;13(7):e12282. doi: 10.1002/clt2.12282 (PMC10345724; doi:10.1002/clt2.12282)
Supplement: Supplementary file 2 — Supporting Information S2 [file CLT2-13-e12282-s001.docx]

**Supporting Information**

**Genetic differences according to onset age and lung function in asthma: a cluster analysis**

Han-Kyul Kim^1†^, Ji-One Kang^1†^, Ji Eun Lim^1^, Tae-Woong Ha^1^, Hae Un Jung^2^, Won Jun Lee^1^, Dong Jun Kim^2^, Eun Ju Baek^2,5^, Ian M Adcock^4^, Kian Fan Chung^4^, Tae-Bum Kim^3^*, Bermseok Oh^1,2,5^*

^1^Department of Biochemistry and Molecular Biology, School of Medicine, Kyung Hee University, Seoul 02447, Korea

^2^Department of Biomedical Science, Graduate School, Kyung Hee University, Seoul 02447, Korea

^3^Department of Allergy and Clinical Immunology, Asan Medical Center, University of Ulsan College of Medicine, Seoul 05505, Korea

^4^The National Heart and Lung Institute, Imperial College, London, UK

^5^Mendel, Seoul 02455, Korea

^†^These authors share co-first authors.

*Corresponding authors:

Bermseok Oh

Department of Biochemistry and Molecular Biology, School of Medicine, Kyung Hee University, Seoul 02447, Korea

Phone: +82 2-961-0617

Mobile: +82 10-8140-1534

E-mail: ohbs@khu.ac.kr

Tae-Bum Kim

Department of Allergy and Clinical Immunology, Asan Medical Center, University of Ulsan College of Medicine, Seoul 05505, Korea.

E-mail: tbkim@amc.seoul.kr

**Supplementary Figures**

**
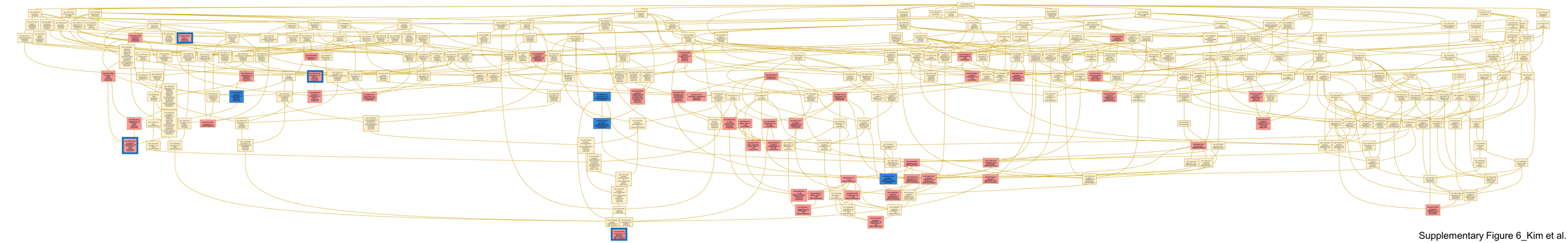
**

**Supplementary Figure S6: Gene-ontology biological process term lists from MAGMA in a directed acyclic graph.** The early-onset asthma^normalLF^ specific GO terms are indicated in red, the early-onset asthma^reducedLF^ specific are indicated in blue, and in case of significant on both sides, there are shown in inner red and blue borders.
