# Supplementary material for: Genetic differences according to onset age and lung function in asthma: A cluster analysis
Source: Clin Transl Allergy. 2023 Jul 14;13(7):e12282. doi: 10.1002/clt2.12282 (PMC10345724; doi:10.1002/clt2.12282)
Supplement: Supplementary file 3 — Supporting Information S3 [file CLT2-13-e12282-s004.docx]

**Supporting Information**

**Genetic differences according to onset age and lung function in asthma: a cluster analysis**

Han-Kyul Kim^1†^, Ji-One Kang^1†^, Ji Eun Lim^1^, Tae-Woong Ha^1^, Hae Un Jung^2^, Won Jun Lee^1^, Dong Jun Kim^2^, Eun Ju Baek^2,5^, Ian M Adcock^4^, Kian Fan Chung^4^, Tae-Bum Kim^3^*, Bermseok Oh^1,2,5^*

^1^Department of Biochemistry and Molecular Biology, School of Medicine, Kyung Hee University, Seoul 02447, Korea

^2^Department of Biomedical Science, Graduate School, Kyung Hee University, Seoul 02447, Korea

^3^Department of Allergy and Clinical Immunology, Asan Medical Center, University of Ulsan College of Medicine, Seoul 05505, Korea

^4^The National Heart and Lung Institute, Imperial College, London, UK

^5^Mendel, Seoul 02455, Korea

^†^These authors share co-first authors.

*Corresponding authors:

Bermseok Oh

Department of Biochemistry and Molecular Biology, School of Medicine, Kyung Hee University, Seoul 02447, Korea

Phone: +82 2-961-0617

Mobile: +82 10-8140-1534

E-mail: ohbs@khu.ac.kr

Tae-Bum Kim

Department of Allergy and Clinical Immunology, Asan Medical Center, University of Ulsan College of Medicine, Seoul 05505, Korea.

E-mail: tbkim@amc.seoul.kr

**Supplementary Figures**

**
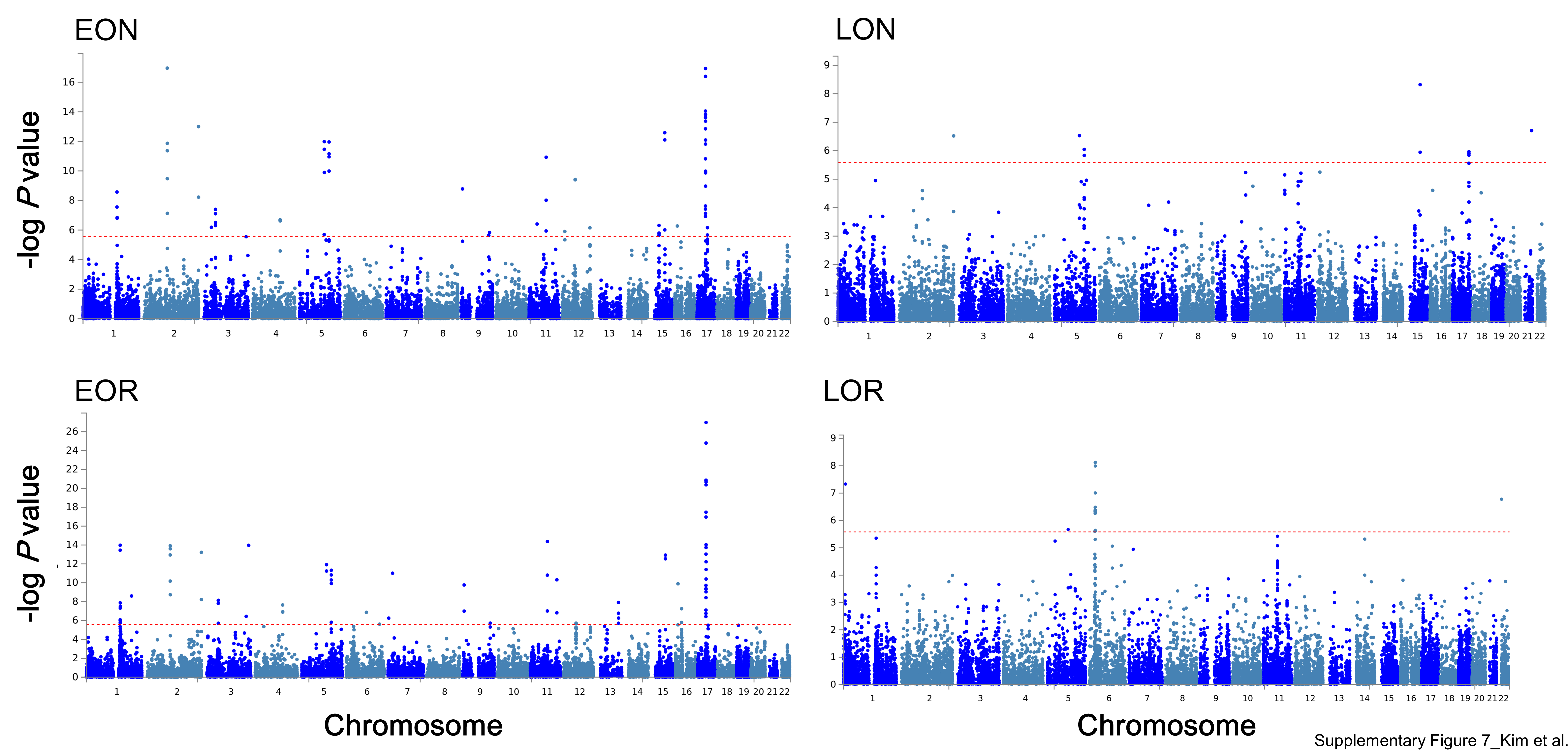
**

**Supplementary Figure S7: P values of associated genes from MAGMA gene-based analysis.** The Manhattan plot was generated based on the gene-based P value calculated using MAGMA for clusters including 19,023 mapped genes. The red dot line indicates the Bonferroni corrected significance level (P < 0.05/19028 = 2.63 × 10^-6^). Cluster-specific signals were in bold and novel signal in red.

**
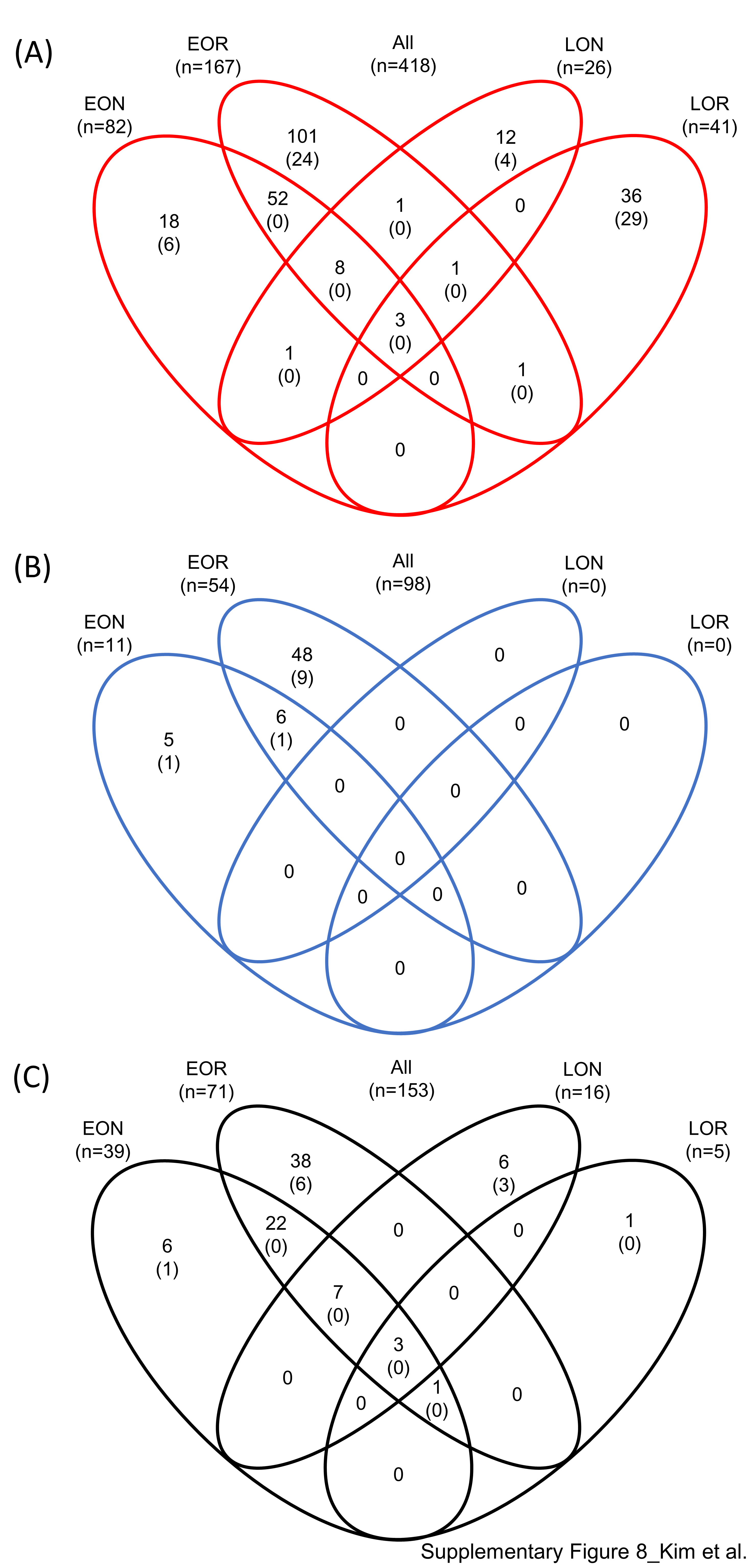
**

**Supplementary Figure S8. Venn diagrams for associated genes and gene sets from MAGMA analysis and SNPs from GWASs.** a) Associated genes, b) associated gene sets, and c) associated lead SNPs. Venn diagrams show the number of overlapped genes and gene sets implicated using MAGMA analysis and that of overlapped SNPs by GWASs. The numbers in parentheses are the number of signals that does not overlap with that of the All asthma group.

**
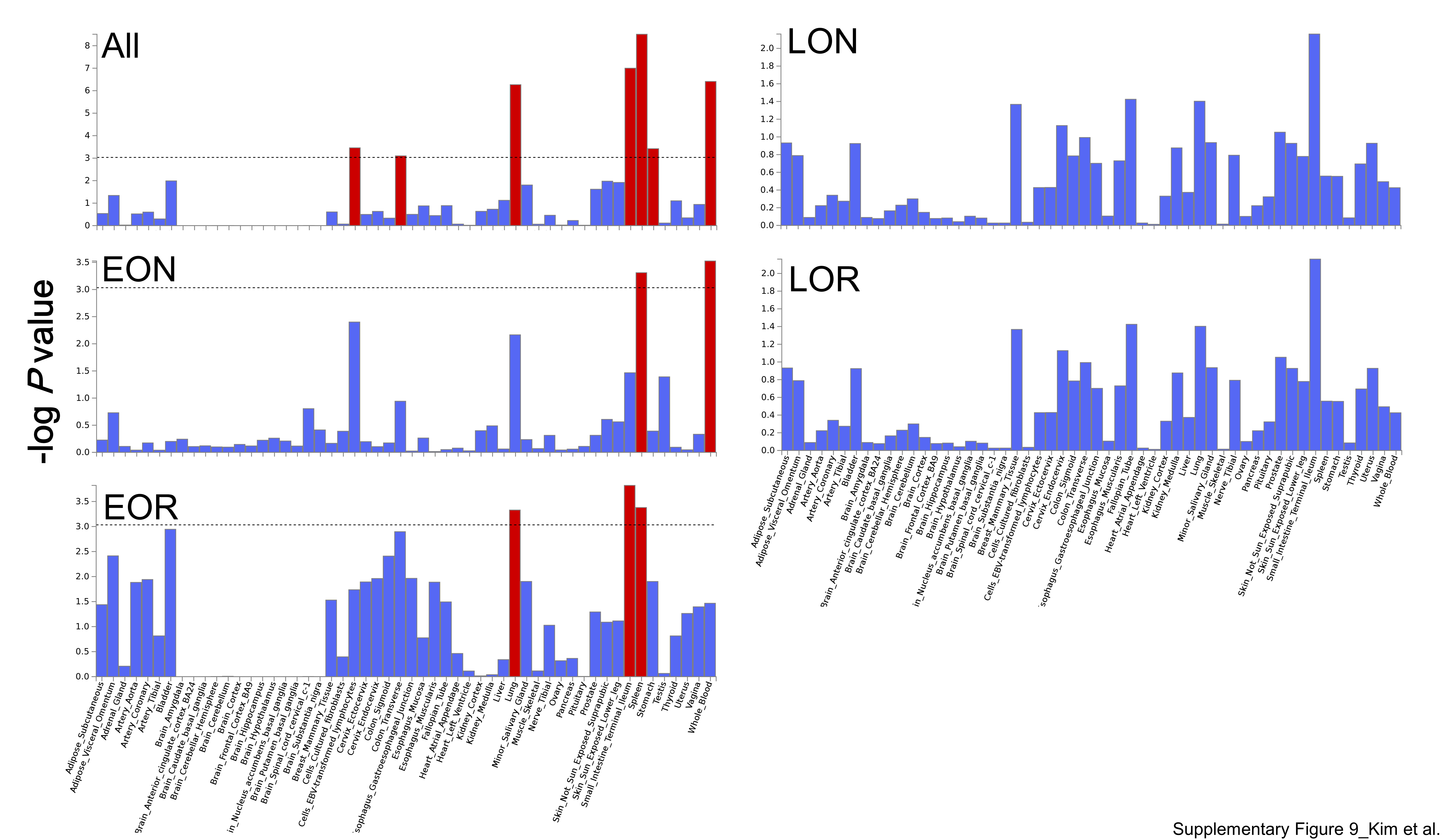
**

**Supplementary Figure S9: Tissue expression profiles of clusters.** Tissue expression profiles were determined using MAGMA gene analysis with the GTEx tissue expression database. Red indicates statistical significance.
